# A Recombination Directionality Factor Controls the Cell Type-Specific Activation of σ^K^ and the Fidelity of Spore Development in *Clostridium difficile*

**DOI:** 10.1371/journal.pgen.1006312

**Published:** 2016-09-15

**Authors:** Mónica Serrano, Nicolas Kint, Fátima C. Pereira, Laure Saujet, Pierre Boudry, Bruno Dupuy, Adriano O. Henriques, Isabelle Martin-Verstraete

**Affiliations:** 1 Instituto de Tecnologia Química e Biológica António Xavier, Universidade Nova de Lisboa, Oeiras, Portugal; 2 Laboratoire Pathogénese des Bactéries Anaérobies, Institut Pasteur, Paris, France; 3 University Paris Diderot, Sorbonne Paris Cité, Paris, France; Max Planck Institute for Terrestrial Microbiology, GERMANY

## Abstract

The strict anaerobe *Clostridium difficile* is the most common cause of nosocomial diarrhea, and the oxygen-resistant spores that it forms have a central role in the infectious cycle. The late stages of sporulation require the mother cell regulatory protein σ^K^. In *Bacillus subtilis*, the onset of σ^K^ activity requires both excision of a prophage-like element (*skin*^*Bs*^) inserted in the *sigK* gene and proteolytical removal of an inhibitory pro-sequence. Importantly, the rearrangement is restricted to the mother cell because the *skin*^*Bs*^ recombinase is produced specifically in this cell. In *C*. *difficile*, σ^K^ lacks a pro-sequence but a *skin*^*Cd*^ element is present. The product of the *skin*^*Cd*^ gene *CD1231* shares similarity with large serine recombinases. We show that CD1231 is necessary for sporulation and *skin*^*Cd*^ excision. However, contrary to *B*. *subtilis*, expression of *CD1231* is observed in vegetative cells and in both sporangial compartments. Nevertheless, we show that *skin*^*Cd*^ excision is under the control of mother cell regulatory proteins σ^E^ and SpoIIID. We then demonstrate that σ^E^ and SpoIIID control the expression of the *skin*^*Cd*^ gene *CD1234*, and that this gene is required for sporulation and *skin*^*Cd*^ excision. CD1231 and CD1234 appear to interact and both proteins are required for *skin*^*Cd*^ excision while only CD1231 is necessary for *skin*^*Cd*^ integration. Thus, CD1234 is a recombination directionality factor that delays and restricts *skin*^*Cd*^ excision to the terminal mother cell. Finally, while the *skin*^*Cd*^ element is not essential for sporulation, deletion of *skin*^*Cd*^ results in premature activity of σ^K^ and in spores with altered surface layers. Thus, *skin*^*Cd*^ excision is a key element controlling the onset of σ^K^ activity and the fidelity of spore development.

## Author Summary

*Clostridium difficile*, a major cause of antibiotic-associated diarrhea, produces resistant spores that facilitate its persistence in the environment including hospitals. *C*. *difficile* transmission is mediated by contamination of gut by spores. Understanding how this complex developmental process is regulated is fundamental to decipher the *C*. *difficile* transmission and pathogenesis. A less tight connection between the forespore and mother cell lines of gene expression is observed in *C*. *difficile* compared to *Bacillus subtilis* especially at the level of the late sigma factor, σ^K^. In *C*. *difficile*, the *sigK* gene is interrupted in most of the strains by a prophage-like intervening sequence, *skin*^*Cd*^, which is excised during sporulation. Contrary to *B*. *subtilis*, *CD1231* encoding the large serine recombinase required for *skin*^*Cd*^ excision, is constitutively expressed and a recombination directionality factor, whose synthesis is detected only in the mother cell, restricts *skin*^*Cd*^ excision to this terminal cell. These two proteins are necessary and sufficient to trigger *skin*^*Cd*^ excision promoting the timely appearance of σ^K^, which in turn switches-on late sporulation events. While several strains of *C*. *difficile* lack a *skin* element, we show that deletion of *skin*^*Cd*^ results in premature σ^K^ activity and in spores with altered surface layers, a property that might be important for host colonization.

## Introduction

Endosporulation is an ancient bacterial cell differentiation process allowing the conversion of a vegetative cell into a mature spore through a series of morphological steps [1, 2]. Many bacilli, clostridia and related organisms form bacterial spores. The spores have the ability to withstand extreme physical and chemical conditions and their resistance properties allow them to survive for long periods in a variety of environments. Spores serve as the infectious vehicle for several pathogens such as *Bacillus anthracis*, *Bacillus cereus* and *Clostridium difficile* [3, 4]. *C*. *difficile* is the main cause of antibiotic-associated diarrhea. Disruption of the intestinal flora caused by antibiotherapy increases the risk to develop a *C*. *difficile* infection. After ingestion, *C*. *difficile* spores germinate in the intestine in the presence of specific bile salts [5]. Then, vegetative forms multiply and produce two toxins, TcdA and TcdB, which are the main virulence factors [6]. These toxins cause enterocyte lysis and inflammation leading to diarrhea, colitis, pseudomembranous colitis or more severe symptoms including bowel perforation, sepsis and death. During the infection process, *C*. *difficile* also forms spores in the gut that are essential for transmission of this strict anaerobe and contribute to the establishment of reservoirs in the environment including the host and hospital settings [7, 8].

Despite the importance of spores in the infectious cycle, our knowledge of the molecular mechanisms underlying spore development in *C*. *difficile* is still scarce. Sporulation has been extensively studied in the model organism *Bacillus subtilis* [9, 10]. At the onset of sporulation, an asymmetric division forms a forespore and a mother cell. A key developmental transition is when the mother cell finishes engulfing the forespore, which becomes fully surrounded by the mother cell. The mother cell maintains metabolic potential in the forespore and contributes to assembly of the spore protective structures and to the release of mature spores. The developmental program of sporulation is mainly governed by the sequential appearance of four cell type-specific sigma factors: σ^F^ in the forespore and σ^E^ in the mother cell control early stages of development, prior to engulfment completion, and are replaced by σ^G^ and σ^K^ following engulfment completion. The main morphological stages of sporulation are conserved among spore-formers, which also share a core of sporulation genes [11, 12]. Nevertheless, recent work has highlighted important differences in the genetic control of sporulation between the aerobic bacilli and the anaerobic clostridia [13–15].

In *C*. *difficile*, the main functions and periods of activity of the sporulation σ factors are largely conserved relative to *B*. *subtilis* [16–18]. In *B*. *subtilis*, several mechanisms including signaling pathways between the two compartments and the architecture of the mother cell- and forespore-specific lines of gene expression, formed by interlocked feed-forward loops (FFLs), converge for the timely activation of the σ factors at specific developmental stages [9, 19]. However, in *C*. *difficile*, the communication between the forespore and the mother cell appears less effective, contributing for a weaker connection between morphogenesis and gene expression [16–18]. Indeed, the activation of the σ^E^ regulon in the mother cell just after asymmetric division, is rigorously dependent on σ^F^ in *B*. *subtilis*, but is partially independent of σ^F^ in *C*. *difficile*. Likewise, the synthesis of the forespore signaling protein SpoIIR, essential for pro-σ^E^ processing, is strictly dependent on σ^F^ in *B*. *subtilis* but partially independent of σ^F^ in *C*. *difficile* [18]. Furthermore, in *B*. *subtilis*, the onset of σ^G^ activity coincides with engulfment completion and requires the activity of σ^E^, while in *C*. *difficile* σ^G^ activity is detected in pre-engulfment sporangia and this early activity is independent of σ^E^ [20, 21]. Finally, several levels of regulation ensure that the activity of σ^K^ in *B*. *subtilis* is restricted to the mother cell following engulfment completion. Firstly, the *sigK* gene is interrupted by an intervening prophage-like element, *skin*^*Bs*^. Secondly, expression of *sigK* and of *spoIVCA* encoding a member of the large serine recombinases (LSRs) superfamily [22] responsible for *skin*^*Bs*^ excision is under the control of σ^E^ and requires the transcriptional regulator SpoIIID [19, 23]. Expression of *spoIIID* is also controlled by σ^E^, but since SpoIIID is auto-regulated [24], a coherent FFL delays expression of the *spoIVCA* and *sigK* genes towards the end of engulfment [19, 25]. Moreover, σ^K^ activity depends on the cleavage of an inhibitory pro-sequence, a step controlled by σ^G^. Finally, σ^K^ directs expression of an anti-sigma factor, CsfB that inhibits σ^E^, thereby promoting transition from σ^E^- to σ^K^-controlled stages in the mother cell [26]. σ^K^ is required for assembly of the spore cortex and the more external coat, the main spore surface structures, as well as for mother cell lysis. The segregation of σ^K^ activity to post-engulfment sporangia in *B*. *subtilis* is explained by multi-level regulation of σ^K^ synthesis and activation. Redundancy ensures fail-safe solutions and increases robustness of the developmental process.

In *C*. *difficile*, σ^K^ is dispensable for cortex biogenesis but is required for the assembly of the spore coat and of the exosporium and for mother cell lysis [17]. *sigK* is interrupted by a *skin*^*Cd*^ element, which is excised during sporulation [27]. The *skin* elements of *B*. *subtilis* and *C*. *difficile* have different sizes and gene content and are inserted at different sites and in opposite orientation indicating that integration into *sigK* has occurred independently during evolution [27]. Previous studies have also shown that some transcription of *sigK* takes place during engulfment [17]. However, σ^K^ of *C*. *difficile* lacks a pro-sequence [27] and accordingly, σ^G^ is dispensable for σ^K^ activation [16–18]. In the absence of this cleavage at the end of engulfment, *skin*^*Cd*^ excision is likely a crucial element in the regulation of σ^K^ activity in *C*. *difficile*. Importantly, given the role of σ^K^ in the assembly of the spore surface layers together with the observation that some strains of *C*. *difficile* lack *skin*^*Cd*^ [27, 28], it is imperative to better understand excision of the element in this organism.

The *CD1231* gene, located within *skin*^*Cd*^, codes for a protein similar to the SpoIVCA recombinase of *skin*^*Bs*^ [27, 29]. Surprisingly, σ^E^ does not control *CD1231* expression in *C*. *difficile* [18, 30], which, as we now show, is expressed constitutively. In this work, we studied the role of *CD1231* in sporulation and in *skin*^*Cd*^ excision. We demonstrated that another factor present in the *skin*^*Cd*^ element, CD1234 encoded by a gene under σ^E^ and SpoIIID control is required for *skin*^*Cd*^ excision but not integration. Thus, CD1234 is a recombination directionality factor that restricts *skin*^*Cd*^ excision to the mother cell. Importantly, we showed that *skin*^*Cd*^ is a key element in controlling the onset of σ^K^ activity, which in turn is important for proper spore morphogenesis and function.

## Results

### Role of *CD1231* in sporulation

**Fig 1:**
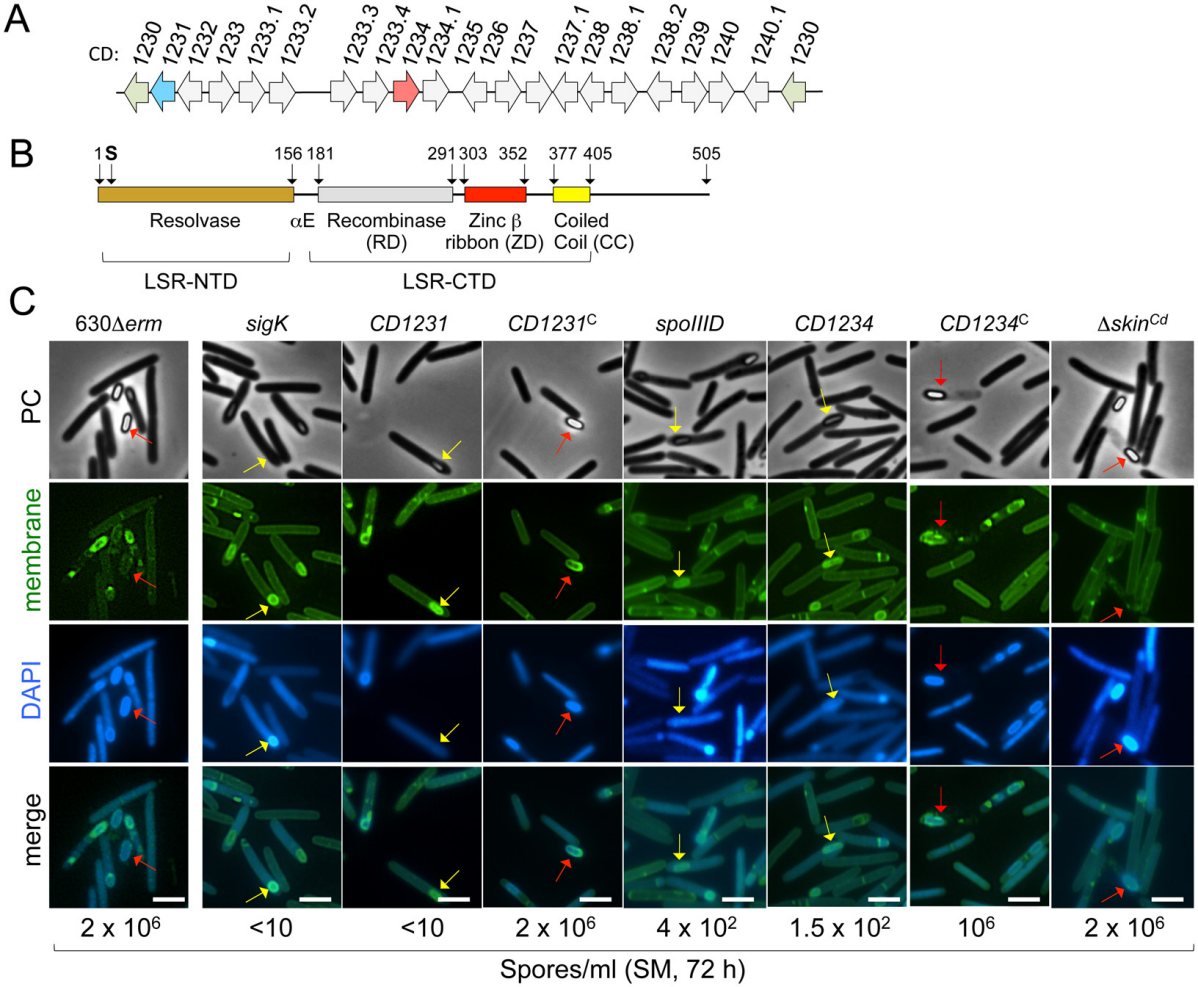
The *skin*^*Cd*^ gene *CD1231*, coding for a serine recombinase, is required for sporulation. **A:** schematic representation of the *sigK*-*skin*^*Cd*^ region of the *C*. *difficile* 630Δ*erm* chromosome. The two halves of the *sigK* gene (5‘ and 3’ part of *CD1230*) are shown in green, and the *CD1231* gene, coding for a protein of the large serine recombinase family is shown in blue. The *CD1234* gene is shown in pink. **B:** domain organization of the CD1231 serine recombinase. The horizontal black line is a linear representation of the amino acid sequence. The three conserved domains identified are color-coded: brown, resolvase domain (PF00239, which forms the N-terminal domain (NTD); grey, recombinase domain (RD; PF07508); red, a zinc ribbon domain (ZD; PF13408); yellow, a coiled-coil (CC) motif. The recombinase domain, the zinc finger and the coiled-coil form the C-terminal domain (CTD). A long α-helix linking the NTD and CTD domains is indicated as αE. The catalytic serine, close to the N-terminal end of the protein, is represented. **C:** cells of the WT strain 630Δ*erm*, the Δ*skin*^*Cd*^, *spoIIID*, *sigK*, *CD1231* and *CD1234* mutants and the complemented strains, CDIP533 and CDIP397, carrying multicopy alleles of *CD1231* (*CD1231*^*C*^) or *CD1234* (*CD1234*^*C*^) expressed under the control of their native promoters were collected after 24 h of growth in liquid SM, stained with the DNA stain DAPI and the membrane dye MTG and examined by phase contrast and fluorescence microscopy. The red arrows point to phase bright spores and the yellow arrows to phase grey spores. Scale bar, 1μm. The titer of heat resistant spores measured for each strain after 72 h of growth in SM is indicated below the panels. The titer of heat resistant spores at 48 h was 0.75 x 10^6^ for strain 630Δ*erm* and for the Δ*skin*^*Cd*^ mutant.

The *skin*^*Cd*^ element of strain 630Δ*erm* inserted into the *sigK* gene contains 19 genes (Fig 1A). *CD1231*, located immediately upstream of the 3´-moiety of *sigK* (*CD1230*), is the unique gene within the *skin*^*Cd*^ element coding for a protein with similarity to large serine recombinases (LSRs) superfamily [22]. The first 300 amino acid residues of CD1231 share 24% identity with SpoIVCA, the recombinase encoded by *skin^Bs^* [31], and 30% identity over its entire length to the SprA protein, responsible for the excision of the *B*. *subtilis* SPβ prophage [32]. CD1231 is also similar to other *C*. *difficile* recombinases associated with conjugative transposons. A domain analysis of CD1231 (Fig 1B) identifies the three main structural domains of LSRs and the motifs that connect them: the N-terminal resolvase domain (LSR-NTD) bearing the conserved catalytic nucleophile (Ser at position 10) and additional catalytic residues, followed by a recombinase domain (RD), a zinc β-ribbon domain (ZD) and a coiled coil (CC) motif (Figs 1B and S1). The RD, the ZD and the CC form the C-terminal domain (LSR-CTD); the NTD and CTD are linked by a long α-helix (αE) while a short linker connects the RD to the ZD [22]. In CD1231 as in other LSRs, the CTD is followed by an extension of variable length, which is mostly α-helical (S1 Fig) [22].

In *B*. *subtilis*, the excision of the *skin*^*Bs*^ element occurs in the mother cell, and creates an intact *sigK* gene, essential for sporulation. Since σ^K^ is required for sporulation in *C*. *difficile* [15], inactivation of *CD1231*, which is probably involved in *skin*^*Cd*^ excision, would cause a block in the process. We constructed a *CD1231* mutant (CDIP526) using the Clostron system (S2 Fig) as well as a complemented strain (CDIP533) carrying *CD1231* under the control of its promoter (pMTL84121-*CD1231*; see below). We then examined the morphology of the strains by phase contrast and fluorescence microscopy after 24 h of growth in sporulation medium (SM), and we tested the efficiency of heat-resistant spore formation at 72 h. The 630Δ*erm* strain produced 2 x10^6^ heat-resistant CFU/ml and phase bright spores, either free or still inside the mother cell, were seen (Fig 1C). In contrast, less than 10 heat resistant CFU/ ml were detected for the *CD1231* mutant. While some phase gray spores were seen in cultures of the mutant at 24 h, free spores were not detected (Fig 1C). Complementation of the *CD1231* mutation restored the wild-type phenotype (Fig 1C). Therefore, inactivation of the *CD1231* gene severely impaired sporulation. The phenotype caused by the *CD1231* mutation phenocopied that imposed by a *sigK* mutation in that phase gray, heat-sensitive spores were formed that often were seen in a angle relative to the long axis of the cell (Fig 1C) [16, 17]. Moreover, as found for a *sigK* mutant, formation of the phase gray spores was not accompanied by loss of viability of the mother cell, as is the case for the wild-type strain [17]. These observations strongly suggest that σ^K^ is inactive in this mutant.

### Constitutive expression of the CD1231 gene

**Fig 2:**
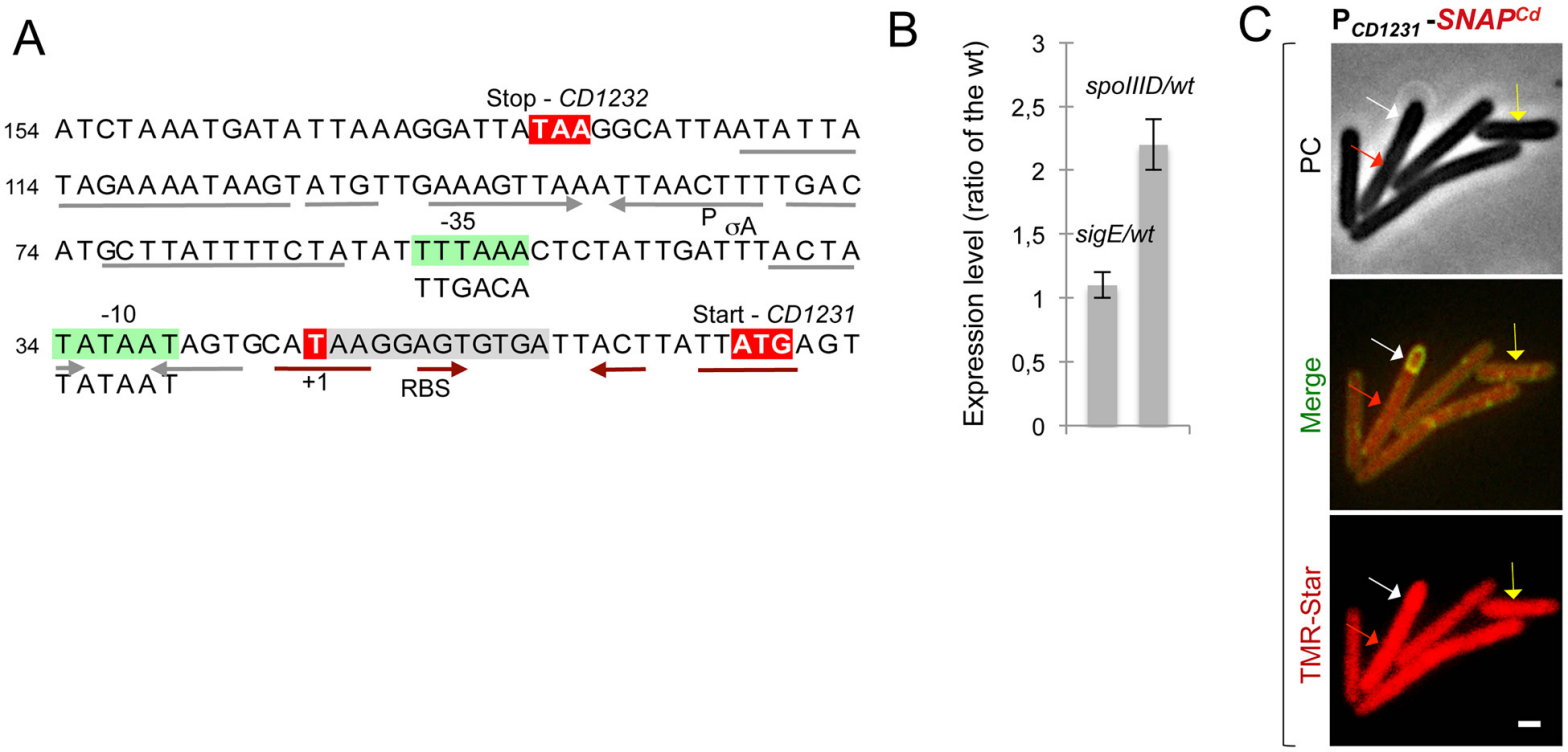
Constitutive expression of the *skin*^*Cd*^ gene *CD1231*. **A:** promoter region of the *CD1231* gene. The mapped transcriptional start sites (+1, red) [34] and the -10 and -35 promoter elements (green boxes) that match the consensus for σ^A^ recognition are indicated. Also represented are the stop codon of *CD1232* and the start codon of *CD1231* (red). Inverted repeats upstream (grey arrows) and downstream (brown) of the +1 position are indicated as well as a possible RBS overlapping the left arm of these repeats. **B:** qRT-PCR analysis of *CD1231* transcription in strain 630Δ*erm*, and in *sigE* or *spoIIID* mutant. RNA was extracted from cells collected 14 h (*sigE* mutant) or 15 h (*spoIIID* mutant) after inoculation in liquid SM. Expression is represented as the fold ratio between the indicated mutants and the wild-type (WT). Values are the average ± SD of two independent experiments. **C**: cells of the *C*. *difficile* 630Δ*erm* strain carrying a P_*CD1231*_-*SNAP*^*Cd*^ transcriptional fusion were collected after 24 h of growth in liquid SM, stained with TMR-Star and the membrane dye MTG, and examined by phase contrast (PC) and fluorescence microscopy. The merged images show the overlap between the TMR-Star (red) and MTG (green) channels. The yellow arrow shows a vegetative cell expressing P_*CD1231*_-*SNAP*^*Cd*^, the white arrow shows expression in the forespore and the red arrow expression in the mother cell. Scale bar, 1 μm.

Expression of the *spoIVCA* gene in *B*. *subtilis* is under the dual control of the mother cell proteins σ^E^ and SpoIIID leading to the restriction of *skin*^*Bs*^ excision to this compartment [19, 33]. However, previous transcriptome studies suggested that σ^E^ or SpoIIID does not control *CD1231* expression in *C*. *difficile* [18, 30]. Moreover, qRT-PCR using RNA extracted from SM cultures of the strain 630Δ*erm*, a *sigE* mutant and a *spoIIID* mutant did not show variations in the level of *CD1231* expression in the *sigE* mutant and only a slight increase in the *spoIIID* mutant relative to the wild-type strain (Fig 2B). In our genome-wide mapping of promoters in strain 630Δ*erm* [34], a transcriptional start site (TSS) was found 21 bp upstream of the *CD1231* start codon and -35 (TTTAAA) and -10 (TATAAT) sequences for σ^A^-dependent promoters are present upstream of this TSS while no consensus for σ^E^ is found (Fig 2A). This suggests that expression of *CD1231* is under the control of σ^A^ and therefore probably not confined to the mother cell. To test this possibility, we constructed a P_*CD1231*_-*SNAP*^*Cd*^ fusion and this fusion was transferred to the 630Δ*erm* strain. Samples of cultures expressing P_*CD1231*_- *SNAP*^*Cd*^ were collected at 24 h of growth in SM, and the cells doubly labeled with the SNAP substrate TMR-Star and the membrane dye MTG. Expression of P_*CD1231*_- *SNAP*^*Cd*^ was detected in 93% of the vegetative cells scored, consistent with the presence of a σ^A^-type promoter. However, expression of P_*CD1231*_-*SNAP*^*Cd*^ was also detected in sporulating cells in both the forespore and the mother cell (78% of the sporangia) (Fig 2C). Thus, in agreement with the absence of a requirement for σ^E^ and SpoIIID for its expression as determined by qRT-PCR, *CD1231* is not a mother cell-specific gene.

### Control of *sigK* transcription and σ^K^ activity by SpoIIID

**Fig 3:**
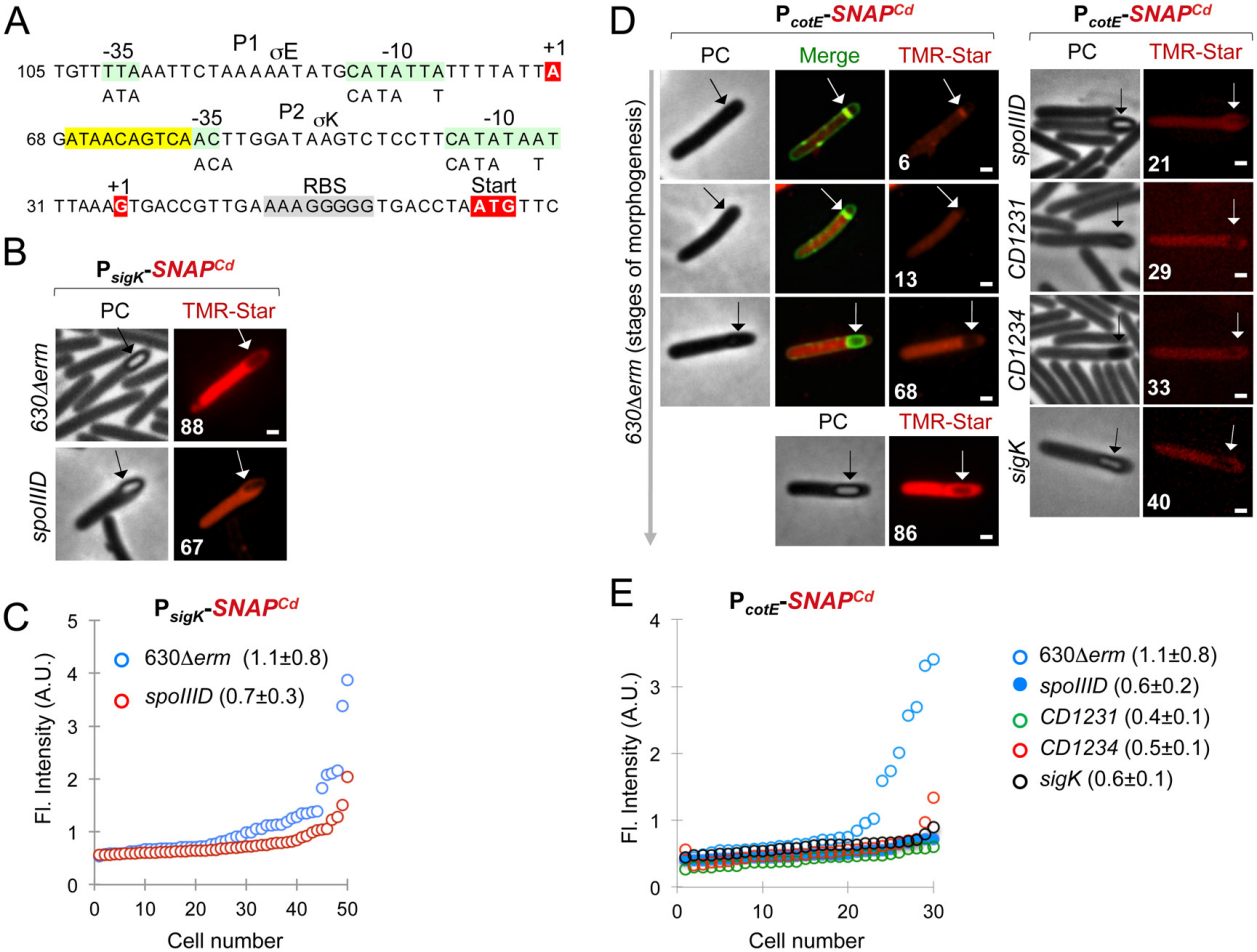
Control of *sigK* expression and of σ^K^ activity. **A:** promoter region of the *sigK* gene. The transcriptional start sites (+1, red) as previously mapped [34], the -10 and -35 promoter elements (green) that match the consensus for σ^E^ or σ^K^ recognition (represented below the sequence), the SpoIIID box (yellow), and the start codon of the *sigK* gene, are indicated. **B:** microscopy analysis of *C*. *difficile* cells carrying a P_*sigK*_-*SNAP*^*Cd*^ fusion in strain 630Δ*erm* and in the *spoIIID* mutant. The cells collected after 24 h of growth in SM were stained with TMR-Star and examined by phase contrast (PC) and fluorescence microscopy. The morphological stage at which *sigK* transcription reaches its maximum, concomitant with the appearance with phase gray spores, is illustrated. The position of the forespore is clearly seen in the PC images. The numbers represent the percentage of cells at the indicated stage that show expression of the reporter fusion. **C:** quantitative analysis of the fluorescence intensity (Fl., in arbitrary units, A.U.) in sporulating cells (as in B) expressing P_*sigK*_- *SNAP*^*Cd*^. The numbers in the legend represent the average fluorescence intensity ± SD (a minimum of 50 cells were scored). **D:** microscopy analysis of cells carrying fusions of the σ^K^-dependent *cotE* promoter to *SNAP*^*Cd*^ in strain 630Δ*erm* and in the *spoIIID*, *CD1231*, *CD1234* and *sigK* mutants. Cells were collected and processed for imaging as indicated in panel B. However, MTG staining was used to visualize the forespore membranes and the stage of sporulation during engulfment; the merged images show the overlap between TMR-star (red) and MTG (green). Note that merged images (MTG/TMR-Star) are not shown whenever the position of the forespore is seen in the PC images. The panels are representative of the expression patterns observed for different sporulation stages, ordered from early to late. For the mutant strains, the morphological stage characteristic of each mutant is shown. The numbers refer to the percentage of cells at the represented stage showing SNAP^Cd^ fluorescence. **E:** quantitative analysis of the fluorescence (Fl.) intensity in the various strains expressing P_*cotE*_-*SNAP*^*Cd*^. The numbers in the panels are the average fluorescence intensity ± SD (30 cells were analyzed). In B and D, the arrows show the position of developing spores. Scale bar, 1 μm.

Previous work indicated that the *skin*^*Cd*^ element is excised from the chromosome only during sporulation [27]. This suggests that a factor is required in addition to CD1231 to trigger excision during sporulation. In a first step to search for this factor, we wanted to establish the requirements for *sigK* transcription and σ^K^ activity. Previous work has shown that SpoIIID is required for sporulation and for the transcription of the *sigK* gene in *C*. *difficile* [16, 18, 30]. Importantly, expression of a *skin*^*Cd*^-less version of the *sigK* gene from a SpoIIID-independent promoter largely bypasses the requirement for SpoIIID for sporulation [30]. While showing that a critical function of SpoIIID in sporulation is to ensure efficient *sigK* expression, this result does not discard a possible role of SpoIIID in *skin*^*Cd*^ excision. Here, we examined the effect of a *spoIIID* mutation on *sigK* transcription using a P_*sigK*_-*SNAP*^*Cd*^ fusion and on the activity of σ^K^ using a fusion of the σ^K^-controlled P_*cotE*_ to *SNAP*^*Cd*^ [17]. Two TSSs have been mapped in the *sigK* promoter region (Fig 3A) [18]. The upstream promoter (P1) matches the consensus for σ^E^ recognition, whereas the downstream promoter (P2) matches the consensus for σ^K^ recognition [18]. Using the consensus of SpoIIID of *B*. *subtilis* [19], a possible SpoIIID binding site is found upstream of P2 and downstream of P1 (Fig 3A). Transcription of *sigK* was detected in the mother cell of the wild-type strain soon after septation and during engulfment [17], but increased following engulfment completion, when it was detected in 88% of the sporangia (Fig 3B). In contrast, transcription of *sigK* was detected in only 67% of the *spoIIID* mutant sporangia in which engulfment was completed (Fig 3B). Moreover, quantification of the fluorescence signal from P_*sigK*_-SNAP^*Cd*^ in those cells revealed a reduction of the signal per sporangia, from 1.1±0.8 arbitrary units (A.U.) in the wild-type strain, to 0.7±0.3 A.U. in the *spoIIID* mutant (Fig 3C). The arrangement of the *sigK* promoter region suggests that the low level of transcription during engulfment may arise from P1 whereas the main period of *sigK* transcription could involve utilization of P2 possibly by σ^E^ first, then by σ^K^, with the assistance of SpoIIID. P2 may also be involved in the late, positive auto-regulation of σ^K^ in cells carrying phase bright spores [17, 18] (see also the Discussion). Thus, following engulfment completion, transcription of *sigK* is reduced, but not abolished, in the absence of SpoIIID both in terms of the number of cells in which transcription is activated and, although a less pronounced effect, in the level of expression per cell. The reduction in *sigK* transcription in the *spoIIID* mutant is in line with earlier results [18, 30].

As a measure of σ^K^ activity, P_*cotE*_-driven production of SNAP^*Cd*^ was detected in the mother cell in 6–13% of the wild-type sporangia during engulfment but increased to 68% just after engulfment completion and was seen in 86% of the sporangia when phase bright spores became visible (Fig 3D). Expression of P_*cotE*_-*SNAP*^*Cd*^ was detected in only 40% of the *sigK* mutant sporangia that reached late stages of morphogenesis to form partially refractile spores, and the average fluorescence signal per sporangia decreased to 0.6±0.1 A.U. (Fig 3E). Importantly, disruption of *spoIIID* or *CD1231* reduced expression of P_*cotE*_-*SNAP*^*Cd*^ to only 21% and 29% of the sporangia that reached late stages of morphogenesis. Moreover, the average fluorescence intensity per cell was of 0.6±0.2 A.U. and 0.4±0.1 A.U. in the *spoIIID* or *CD1231* mutant, respectively as compared to 1.1±0.8 A.U. for the wild-type strain (Fig 3D and 3E). Thus, disruption of *sigK*, *spoIIID*, or *CD1231* reduced expression of the P_*cotE*_-*SNAP*^*Cd*^ fusion approximately to the same extent. In any event, the increase in σ^K^ activity following engulfment completion is not seen in *CD1231* and *spoIIID* mutants, compatible with a role for CD1231 and SpoIIID in the control of σ^K^ activity. This strongly suggests that SpoIIID may play a role in *skin*^*Cd*^ excision.

### The cell type-specific excision of *skin*^Cd^ requires SpoIIID

**Fig 4:**
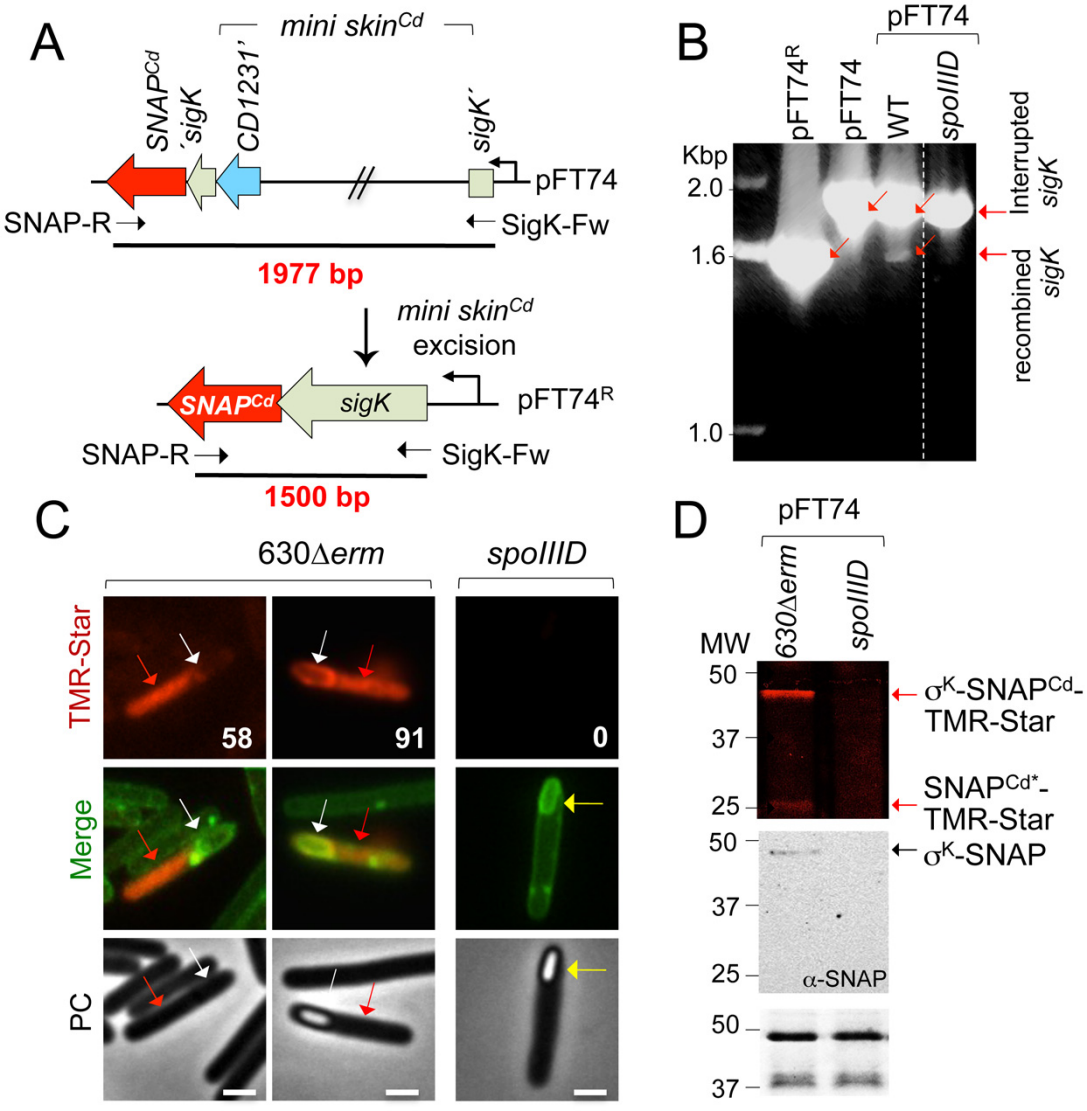
Time of excision of a mini-*skin*^*Cd*^ element as detected by production of a σ^K^-SNAP^Cd^ fusion protein. **A:** schematic representation of plasmid pFT74, containing the *SNAP*^*Cd*^ reporter fused in frame to the 3´-end of *sigK*. The *sigK* gene is interrupted by a mini-*skin*^*Cd*^ element carrying the 3´end of the *CD1231* gene (blue). When *skin*^*Cd*^ excision occurs to form pFT47^R^, σ^K^-SNAP^Cd^ is produced. The size of the inserts before and after recombination is indicated. The primers used for PCR analysis are indicated. **B:** analysis of *skin*^*Cd*^ excision by PCR using the primer pair indicated in panel A and DNA extracted from cultures of the strain 630Δ*erm*, and the *spoIIID* mutant after 24 h of growth in SM. The red arrows point to the recombined *sigK* and the interrupted gene. pFT74 and pFT74^R^ were used as controls for non-recombined and recombined *sigK*, respectively. **C:** fluorescence microscopy analysis of sporulating cells producing σ^K^-SNAP^Cd^ in the strain 630Δ*erm* and in the *spoIIID* mutant. Cells grown in SM were collected at 24 h and stained with the membrane dye MTG and TMR-Star. The red arrows point to the mother cell, and the white arrow to the developing spore. The yellow arrow shows the position of a spore in a *spoIIID* sporangium. The numbers refer to the percentage of cells at the represented stage showing production of the fusion. Scale bar, 1 μm. **D:** accumulation of σ^K^-SNAP^Cd^ in extracts produced from sporulating cells of the WT and *spoIIID* mutant. The cells were collected from SM cultures 24 h after inoculation, labeled with the TMR-Star substrate. Proteins in whole cell lysates were resolved by SDS-PAGE, visualized by fluoroimaging or subject to immunoblot analysis with anti-SNAP antibodies. A section of the corresponding Coomassie-stained gel is shown as a loading control. The arrows indicate the position of the TMR-Star-labeled σ^K^-SNAP^Cd^ fusion (expected size 47 kDa). The asterisk points to a possible degradation product of σ^K^-SNAP^*Cd*^ (SNAP^Cd^*) of about 25 kDa. The position of molecular mass markers (in kDa) is shown on the left side of the panels.

To examine the time and requirements of *skin*^*Cd*^ excision, we devised an assay to monitor reconstitution of a functional *sigK* gene in *C*. *difficile*. We previously described a plasmid, pFT38, carrying a *sigK* gene disrupted by a mini-*skin*^*Cd*^ element bearing a deletion of all the *skin*^*Cd*^ genes except *CD1231* [17]. We modified this plasmid in order to create a translational fusion between the C-terminal moiety of σ^K^ and *SNAP*^*Cd*^ and to remove the 5´-end of *CD1231* (*i*.*e*., only the chromosomal *CD1231* is functional) (Fig 4A). This plasmid, pFT74, was introduced in strain 630Δ*erm* and in the *spoIIID* mutant. Excision through recombination involving sequences at the ends of the mini-*skin*^*Cd*^ element reconstitutes the *sigK* gene (Fig 4A). The recombined *sigK* in pFT74 named pFT74^R^ was first detected by PCR using an oligonucleotide hybridizing to the 5’ moiety of the *sigK* gene and a second in the *SNAP*^*Cd*^ gene: a fragment of 1500 bp is expected upon mini-*skin*^*Cd*^ excision instead of 1977 bp for pFT74 (Fig 4A). The 1500 bp PCR fragment was detected in strain 630Δ*erm* but not in the *spoIIID* mutant (Fig 4B) indicating that SpoIIID is necessary for mini-*skin*^*Cd*^ excision. This is also the case for the chromosomal copy of the *skin*^*Cd*^ element as described below.

To gain further insight into the time of excision relative to the course of spore morphogenesis, we monitored production of the σ^K^-SNAP^Cd^ translational fusion formed after mini-*skin*^*Cd*^ excision at the single cell level by fluorescence microscopy (Fig 4C). Production of σ^K^-SNAP^Cd^ was detected only in the mother cell in 58% of the wild-type sporangia in which spores were not yet discernible in the mother cell by phase contrast microscopy but that were close to or just after engulfment completion as judged from the MTG staining pattern (membranes almost fused or fused) (Fig 4C). However, σ^K^-SNAP^Cd^ was detected in 91% of the sporangia in which partially phase bright or phase bright spores were visible by phase contrast microscopy (Fig 4C). This parallels the pattern of *sigK* transcription and σ^K^ activity [17] and suggests that σ^K^ is active as soon as it is produced after *skin*^*Cd*^ excision. In contrast, no accumulation of σ^K^-SNAP^Cd^ was detected in the *spoIIID* mutant (Fig 4C) even if the *sigK* gene remains expressed in this mutant (Fig 3A). σ^K^-SNAP^Cd^ (47 kDa) was detected by Western blotting using anti-SNAP antibodies and by fluorimaging in crude extracts of strain 630Δ*erm* but not in extracts prepared from the *spoIIID* mutant (Fig 4D). These results strongly suggest that SpoIIID is required for *skin*^Cd^ excision in *C*. *difficile* as also observed for *B*. *subtilis*. Moreover, since the main period of *sigK* transcription, σ^K^ accumulation and σ^K^ activity appear to coincide during the course of morphogenesis, *skin*^Cd^ excision and *sigK* transcription seem to concur to delay the onset of σ^K^ activity.

### CD1234 is a mother cell-specific *skin*^Cd^ gene

**Fig 5:**
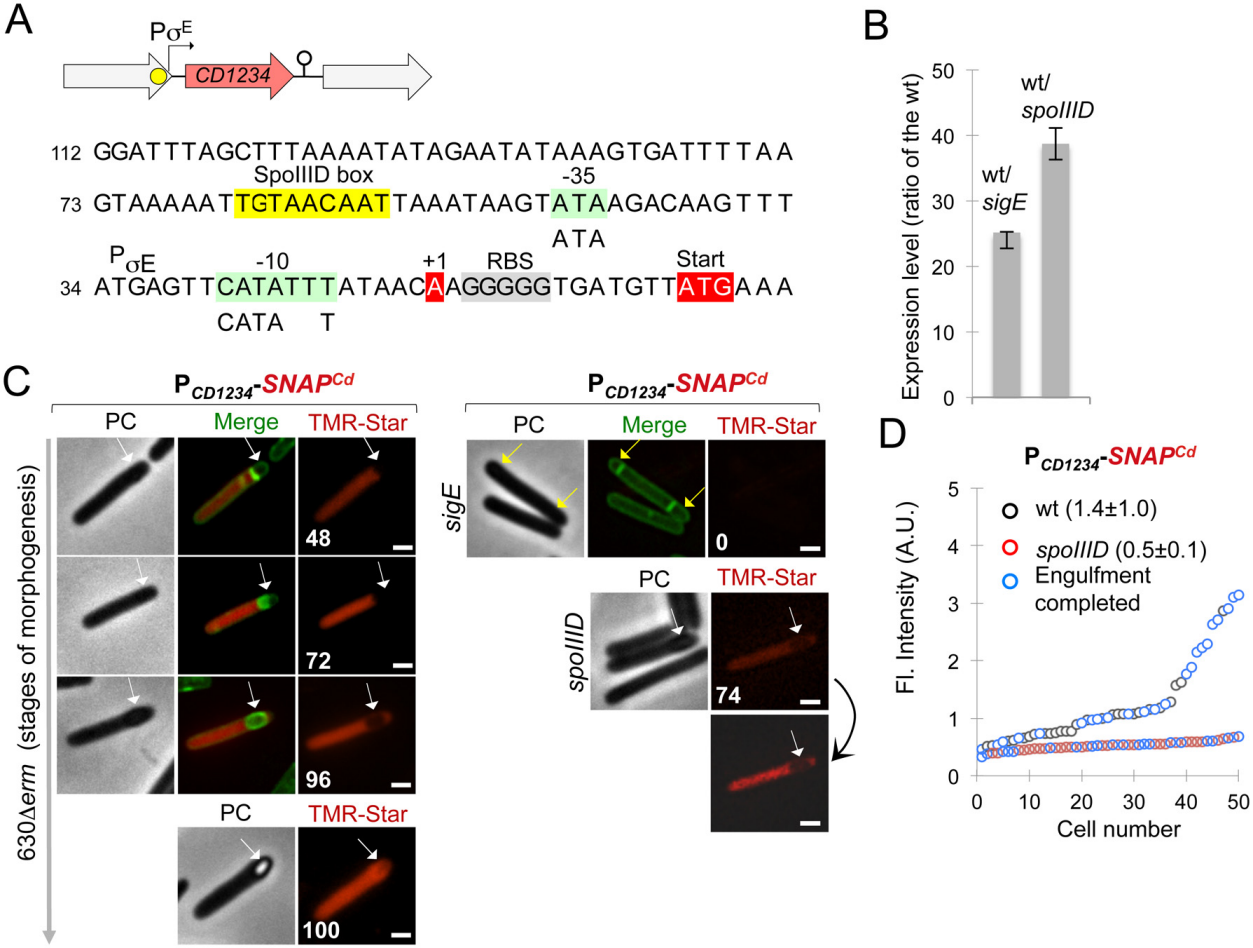
*CD1234* is a mother cell-specific gene. **A**: the panel represents the region of the *CD1234* gene within the *skin*^*Cd*^ element (top) and the sequence of its promoter region (bottom). The transcriptional start site (+1, red), the -10 and -35 promoter elements (green) that match the consensus for the σ^E^ binding (represented below the sequence), and a putative SpoIIID binding site (yellow) are represented. **B**: qRT-PCR analysis of *CD1234* transcription in strain 630Δ*erm* (WT), and in *sigE* or *spoIIID* mutant. RNA was extracted from cells collected 14 h (*sigE* mutant) and 15 h (*spoIIID* mutant) after inoculation in liquid SM. Expression is represented as the fold ratio between the WT strain and the indicated mutants. Values are the average ± SD of two independent experiments. **C:** microscopy analysis of cells of the 630Δ*erm* strain and of the *sigE* and *spoIIID* mutants carrying a P_*CD1234*_-SNAP^Cd^ transcriptional fusion. The cells were collected 24 h after inoculation in liquid SM, stained with TMR-Star and the membrane dye MTG, and examined by phase contrast (PC) and fluorescence microscopy. Merged images (MTG/TMR-Star) are not shown whenever the position of the forespore is clearly seen in the phase contrast images. The panels are representative of the expression patterns observed for different stages of sporulation, ordered from early to late. For the mutant strains, the morphological stage characteristic of each mutant is shown. The numbers in the panels refer to the percentage of cells at the represented stage showing SNAP^Cd^ fluorescence. The white arrows show the position of the developing spore in WT or *spoIIID* sporangia, and the yellow arrows the two-forespore compartments characteristic of a *sigE* mutant (disporic phenotype). Note that the TMR-Star panel for the *spoIIID* mutant is shown repeated with enhanced contrast (the two panels linked by a curved arrow) so that the lack of signal in the forespore is clearly seen. Scale bar, 1 μm. **D**: quantitative analysis of the fluorescence intensity in sporulating cells of the strains shown in C. The numbers in the legend represent the average ± SD of fluorescence intensity (50 cells were scored in each case) in the WT or *spoIIID* mutant. No difference was observed for sporangia before (black symbol in the curve for the WT and red symbols in the curve for the *spoIIID* mutant) or after engulfment completion (blue symbols in both curves).

Given that *skin*^Cd^ excision did not take place in vegetative cells or in the forespore in spite of *CD1231* expression in these cells and the suspected role of SpoIIID in controlling σ^K^ activity via *skin*^*Cd*^ excision, we inferred that a factor probably encoded within *skin*^*Cd*^ and produced under the joint control of σ^E^ and SpoIIID could modulate CD1231 synthesis and activity. Among the 19 *skin*^*Cd*^ genes, only *CD1234* (Fig 5A) was down-regulated in a *sigE* and in a *spoIIID* mutant in transcriptome analyses [18, 30]. *CD1234* codes for a small protein of 72 amino acids, with a predicted pI of 5.5 and no significant similarity to proteins found in databases. We confirmed by qRT-PCR using RNA extracted from SM cultures that *CD1234* expression decreased 25-fold and 40-fold in a *sigE* and in a *spoIIID* mutant, respectively as compared to the wild-type strain (Fig 5B). We mapped a TSS 14 bp upstream of the start codon of *CD1234*, and a consensus sequence for σ^E^ recognition was detected upstream of this TSS [18]. Using the SpoIIID consensus sequence of *B*. *subtilis* [19], we also identified a putative SpoIIID binding motif (TGTAACAAT) centered 46 bp upstream of the *CD1234* TSS (Fig 5A) in agreement with the positive control of *CD1234* expression by SpoIIID.

To examine the compartment and time of *CD1234* expression during sporulation, we constructed a P_*CD1234*_-*SNAP*^*Cd*^ transcriptional fusion. This fusion was transferred by conjugation into the 630Δ*erm* strain and the *sigE* or *spoIIID* mutant. In the wild-type strain, SNAP production confined to the mother cell, was detected in 48% and 72% of the cells just after asymmetric division and during engulfment, respectively and persisted until late stages in development, when phase bright spores were seen (Fig 5C). SNAP^Cd^ production was eliminated by mutation of *sigE* (Fig 5C) while the average intensity of the SNAP^Cd^-TMR signal decreased from 1.4±1 in the 630Δ*erm* strain to 0.5±0.1 in a *spoIIID* mutant (Fig 5D). Together, these results indicate that *CD1234* is a mother cell-specific gene expressed under the joint control of σ^E^ and SpoIIID.

### The *CD1234* gene is required for sporulation

To investigate the role of CD1234 in sporulation, we constructed a *CD1234* mutant, CDIP396, using the Clostron system (S2 Fig). A complemented strain, CDIP397, carrying the *CD1234* gene expressed under the control of its native promoter was also constructed. We examined the morphology of the *CD1234* mutant using phase contrast and fluorescence microscopy. Some phase bright or partially phase bright spores were present in cultures of the *CD1234* mutant but free spores were not detected. The mutant formed only 1.5 x 10^2^ heat-resistant spores/ml of culture at 72 h of growth in SM (i.e. 10^4^ less than the wild-type) (Fig 1C). Importantly, the wild-type phenotype was restored in the complemented strain (Fig 1C). Thus, inactivation of the *CD1234* gene strongly impaired sporulation. Nevertheless, the *CD1234* mutant like the *spoIIID* mutant was not as severely affected in sporulation as the *sigE* and *sigK* mutants [16, 17] or *CD1231* mutant (Fig 1C).

### CD1231 and CD1234 are required for σ^K^ activity

Since both CD1231 and CD1234 are likely required for *skin*^*Cd*^ excision, a prerequisite for σ^K^ activation, we tested the impact of *CD1231* and *CD1234* inactivation on the expression of σ^K^ or σ^E^ targets using qRT-PCR (Table 1). We extracted RNA from strain 630Δ*erm*, the *CD1231* and *CD1234* mutants and the complemented strains after 24 h of growth in SM, a time where σ^K^ target genes are highly expressed [18]. As expected, expression of *CD1231* and *CD1234* was strongly reduced in the *CD1231* and *CD1234* mutants, respectively (Table 1). The expression of three σ^E^ targets (*spoIIIAA*, *spoIVA*, *spoIIID*) was not significantly altered in the *CD1231* and *CD1234* mutants as compared to the wild-type strain. In sharp contrast, expression of six σ^K^ target genes strongly decreased in the *CD1231* or *CD1234* mutant compared to the wild-type strain (Table 1), as observed previously for a *sigK* mutant [18]. *CD1231* and *CD1234* were about 10-fold more expressed in the *CD1231* (pMTL84121-*CD1231*) and *CD1234* (pMTL84121-*CD1234*) strains than in 630Δ*erm* (Table 1), and expression of the σ^K^ target genes was fully or partially restored in these strains. Accordingly, expression of a P_*cotE*_-*SNAP*^*Cd*^ fusion decreased in a *CD1231* mutant compared to strain 630Δ*erm* as described above and was reduced in the *CD1234* mutant. Only 33% of the sporangia that reached late stages of development expressed the fusion when *CD1234* is inactivated compared to 86% for the wild-type strain (Fig 3D). Moreover, the average intensity of the fluorescence signal decreased from 1.1±0.8 A.U. (WT) to 0.5±0.1 (*CD1234*), similar to the intensity seen for the *CD1231* mutant (Fig 3E). In conclusion, expression of σ^K^-dependent but not of σ^E^-dependent genes requires CD1231 and CD1234 as expected for proteins involved in *sigK* reconstruction through *skin*^*Cd*^ excision.

**Table 1:** Effect of *CD1231* or *CD1234* inactivation on the expression of σ^K^ or σ^E^ targets.

	Fold change			
Gene	630Δ*erm*/ *CD1231*	630Δ*erm*/ *CD1231*^C^	630Δ*erm*/ *CD1234*	630Δ*erm*/ *CD1234* ^C^
*CD1231*	11.5 +/-1.5	0.1 +/-0.01	1.24 +/-0.02	1.3 +/-0.03
σ^**E**^ **targets**				
*spoIIIAA*	1.3 +/-0.3	ND	0.85 +/- 0.25	ND
*spoIVA*	2.9 +/-1	ND	1.7 +/- 0.1	ND
*spoIIID*	2.45 +/-0.5	ND	1.3 +/- 0.7	ND
*sigK*	167 +/-12	2.55 +/- 0.1	206 +/-32	1.9 +/- 0.1
*CD1234*	1.85 +/- 0.05	2 +/- 0.1	545 +/-140	0.11 +/-0.01
σ^**K**^ **targets**				
*sleC*	95 +/- 19	4.75 +/-1.25	86 +/- 20	3.75 +/- 0.25
*cotBC*	2045 +/- 49	9 +/- 1	1650 +/-430	1 +/- 0.4
*bclA1*	88 +/- 4.5	2.6 +/- 1.3	53 +/- 6	2.8 +/- 1.1
*cotE*	149 +/-5	8.5 +/- 3	89 +/- 8	6 +/-0.2
*CD1133*	31 +/- 2	2.7 +/- 0.8	30 +/-1	2.2 +/-0.8
*CD3580*	70 +/-15	2.9 +/- 0.9	50 +/-5	3 +/- 0.5

Total RNAs were extracted from *C*. *difficile* 630Δ*erm* strain, the *CD1231* and *CD1234* mutants, and the complementation strains *CD1231*^C^ (*CD1231* mutant with pMTL84121-*CD1231*) and *CD1234*^C^ (*CD1234* mutant with pMTL84121-*CD1234*) after 24 h of growth in SM medium. After reverse transcription, specific cDNAs were quantified by qRT-PCR using the DNApolIII gene for normalization. The results presented correspond to the mean of at least two independent experiments.

### *skin*^*Cd*^ excision during growth and sporulation requires both CD1231 and CD1234

**Fig 6:**
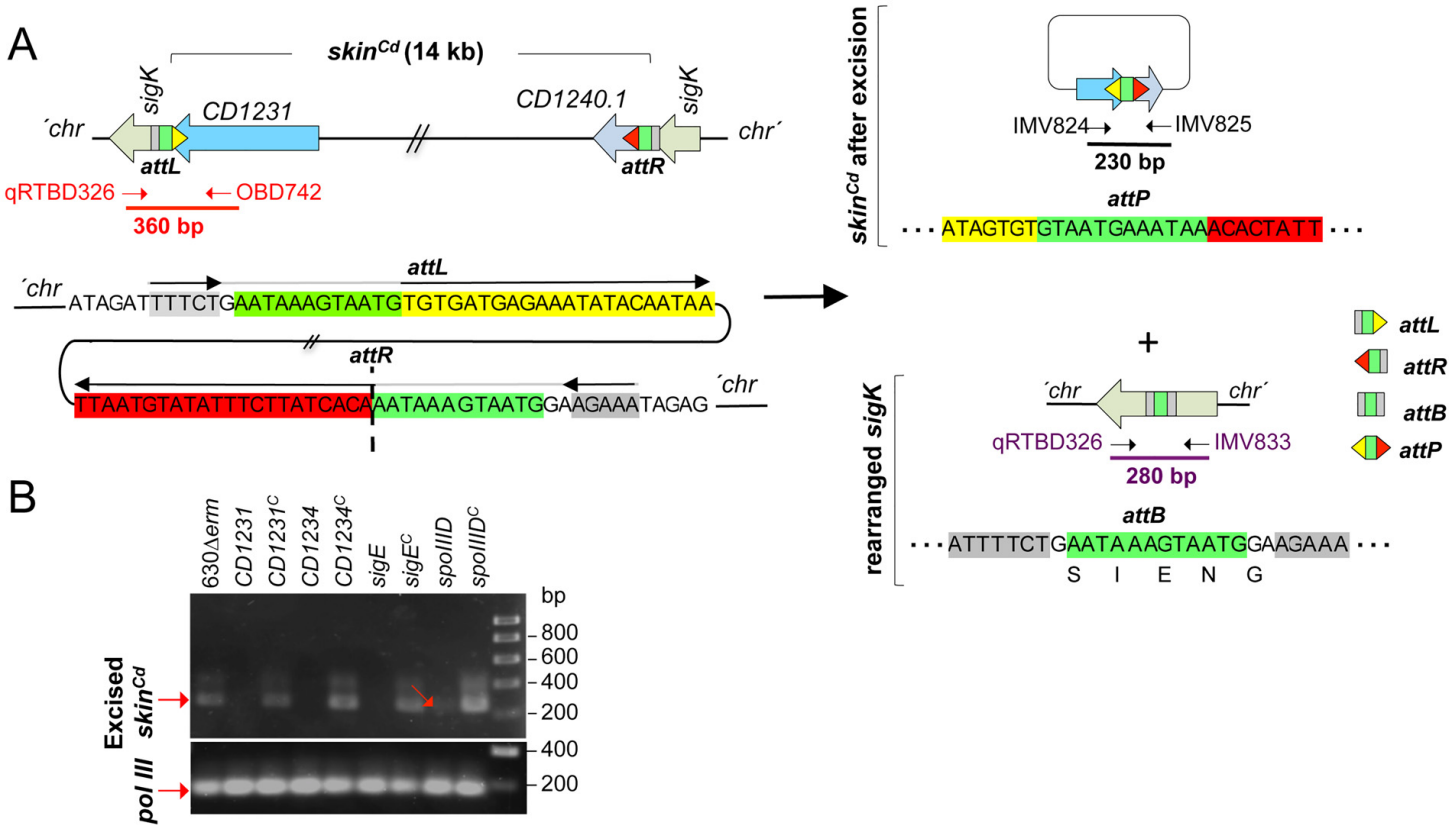
Requirements for *skin*^*Cd*^ excision in *C*. *difficile*. **A:** schematic representation of *skin*^*Cd*^ excision from the 630Δ*erm* chromosome and of the recombination products derived from the process. The *attL* (the half-sites are represented by the green box and the yellow triangle) and *attR* (the green box and the red triangle) in the chromosome, *attP* in the excised *skin*^*Cd*^ (half-sites correspond to yellow and red triangles) and *attB* in the chromosome (green and grey boxes) are represented. The horizontal arrows represent inverted repeats. The vertical dashed line represents the point of junction of the sequenced following recombination. The oligonucleotides used to amplify the 5’ junction of *skin*^*Cd*^ (OBD742-qRTBD326), the reconstructed *sigK* gene (qRTBD326-IMV833) and the circularized *skin*^*Cd*^ (IMV824-IMV825) are indicated, as well as the expected size of the respective products. **B:** detection of *skin*^*Cd*^ excision in different strains by PCR using primers IMV824 and IMV825 and DNA extracted from the indicated strains grown in liquid SM medium for 24 h. *CD1231*^*C*^ denotes the *CD1231* mutant complemented with pMTL84121-*CD1231* and *CD1234*^*C*^ the *CD1234* mutant complemented with pMTL84121-*CD1234*. *sigE*^C^ and *spoIIID*^C^ correspond to the *sigE* or *spoIIID* mutant complemented with pMTL84121-*sigE* or pMTL84121-*spoIIID*, respectively.

The temporal control of *skin*^*Cd*^ excision and its confinement to the terminal mother cell may thus require the σ^E^- and SpoIIID-controlled gene, *CD1234*. We tested excision of the chromosomal *skin*^*Cd*^ in strain 630Δ*erm* and in the *CD1231*, *CD1234*, *sigE* and *spoIIID* mutants (Fig 6). In *B*. *subtilis*, *skin*^*Bs*^ excision occurs within two 5 bp inverted repeats that flank an imperfect 21 bp repeat [35]. In *C*. *difficile*, excision is expected to occur by means of a recombination event involving *attL* and *attR* (by analogy with the sequences involved in phage excision) at the left and right ends of *skin*^*Cd*^ (Fig 6A) [27]. As in *B*. *subtilis*, *attL* and *attR* consist of two half-sites formed by a 5 bp inverted repeat external to a longer 22 bp imperfect inverted repeat (Fig 6A) and of two conserved 12 bp motifs, one in *attL* and one in *attR*, within which recombination take place (in green in Fig 6A). The intervening DNA is excised as a circular molecule carrying *attP* (by analogy with sequences responsible for phage integration), leaving behind a chromosomal *attB* site (analogous to prophage insertion sequences in the bacterial chromosome) (Fig 6A).

The excised circular element obtained after excision of *skin*^*Cd*^ can be monitored by PCR using primers annealing upstream and downstream of *attP* (Fig 6A). During sporulation, *skin*^*Cd*^ excision was detected in strain 630Δ*erm* (Fig 6B, lane 1), but not in the *CD1231* (lane 2), *CD1234* (lane 4) and *sigE* (lane 6) mutants. A very faint band corresponding to the excised *skin*^*Cd*^ was detected in the *spoIIID* mutant (lane 8, red arrow and red dot). Plasmids bearing the disrupted chromosomal genes restored *skin*^*Cd*^ excision to all mutants (Fig 6B, lane 3, 5, 7, 9). Thus, *skin*^*Cd*^ excision during sporulation requires both CD1231 and CD1234. Moreover, the results confirm the key role of SpoIIID and σ^E^ in *skin* excision likely through their control of the cell type-specific production of CD1234.

To test whether expression of *CD1234* could result in *skin*^*Cd*^ excision during vegetative growth, we constructed a plasmid carrying *CD1234* under the control of an ATc-inducible promoter (pDIA6103-P_tet_*CD1234*). This plasmid or the empty vector pDIA6103 were introduced into strain 630Δ*erm* and the *sigE*, *spoIIID*, *CD1231* and *CD1234* mutants. The resulting strains were grown for 4 h in TY medium. Following induction of *CD1234* expression, the cells were either plated onto BHI or harvested for DNA extraction. qPCR was then performed with 2 primer pairs, one corresponding to DNApolIII as a control and the second to *sigK* on both sides of *attB* for the detection of *skin*^*Cd*^ excision. The ΔCt (Ct_*sigK*_-Ct_*polIII*_) was determined for each strain carrying either pDIA6103 or pDIA6103-P_tet_*CD1234*. The ΔCt was >10 for all strains containing pDIA6103. Interestingly, the ΔCt was reduced to < 1 for all strains containing pDIA6103-P_tet_*-CD1234* with the exception of the *CD1231* mutant where a ΔCt of 18 was observed, as was also the case for the strain *CD1231* (pDIA6103) (Table 2). In parallel, chromosomal DNA was extracted from 8 independent clones obtained after seeding BHI plates with samples from the cultures of the different strains carrying pDIA6103-P_tet_*CD1234*. For each clone, we tested the presence of *skin*^*Cd*^ in the chromosome by PCR amplification of the 5’ junction of the *skin* (*attL*) using one oligonucleotide located in the 3’ part of *CD1231* and the second in *sigK* (Fig 6A). While the *skin*^*Cd*^***/***chromosome junction was detected in only 1 out of 8 clones tested for strains 630Δ*erm*, *sigE*, *spoIIID* or *CD1234*, this junction was amplified for the 8 clones of the *CD1231* mutant (Table 2). This confirmed that *skin*^*Cd*^ was excised after induction of *CD1234* expression during growth of the wild-type strain and of the *sigE*, *spoIIID* and *CD1234* mutants. In contrast, *skin*^*Cd*^ remained integrated in the *CD1231* mutant under similar conditions.

**Table 2:** Excision of *skin*^*Cd*^ during growth in strains producing *CD1234* under P_*tet*_ control.

	ΔCt = Ct_*sigK*_-Ct_*polIII*_		Detection of *skin*^Cd^ junction by PCR
Strain	pDIA6103	pDIA6103-P_tet_-*CD1234*	
630Δ*erm*	10.9+/-0.5	0.8+/-0.4	0/8
*sigE*::*erm*	17.9+/-0.5	0.6+/-0.5	1/8
*spoIIID*::*erm*	16.3+/-0.15	0.85+/-0.35	1/8
*CD1231*::*erm*	18.3+/-0.25	18+/-1	8/8
*CD1234*::*erm*	17.9+/-0.25	0.65+/-0.35	1/8

Strains 630Δ*erm*, *sigE*::*erm*, *spoIIID*::*erm*, *CD1234*::*erm* and *CD1231*::*erm* containing either pDIA6103 or pDIA6103-P_tet_-*CD1234* were grown in TY medium for 4 h at which time ATc (100 ng/ml) was added. After 2 h of induction, cells were serially diluted and plated on BHI or collected and DNA extracted. qPCR was performed on with 2 primer pairs: one corresponding to DNApolIII as a control and the second to *sigK* on both sides of the *skin* insertion site (qRTBD325-qRTBD326). Chromosomal DNA extracted from 8 independent clones obtained after plating the cultures on BHI was used to amplify the 5’ junction of the *skin* using one primer in *sigK* (qRTBD326) and one in *CD1231* (OBD742) (See Fig 6A). The number of positive clones among the eight tested is indicated.

In conclusion, these results indicate that during *C*. *difficile* growth: i) excision occurs if and only if *CD1234* is produced; ii) under these conditions *skin*^*Cd*^ excision is independent of σ^E^ and SpoIIID; and iii) excision is absolutely dependent on the *skin*^*Cd*^ CD1231 recombinase, even if *CD1234* is induced. Together, these results indicate that both CD1231 and CD1234 are necessary for *skin*^*Cd*^ excision in *C*. *difficile*.

### Evidence that CD1231 and CD1234 directly interact

Since *CD1231* was not specifically transcribed during sporulation and its expression was not altered in a *CD1234* background (Table 1), we reasoned that CD1234 could post-transcriptionally control the synthesis or activity of CD1231. The integration reaction catalyzed by the LSRs is unidirectional, in that excision often requires an additional recombination directionality factor (RDF) that modulates the LSR activity by direct protein-protein interactions [22, 36, 37]. We therefore tested whether CD1231 and CD1234 could interact using pull-down assays. Whole cell extracts prepared from *E*. *coli* BL21(DE3) strains producing separately CD1234-His_6_ and CD1231-*Strep* or co-producing the two proteins under the control of P_T7*lac*_ were prepared. None of the proteins was detected by Coomassie staining, but they were detected by immunoblotting with antibodies to their C-terminal tags (S4A and S4B Fig). The extracts were then incubated with Ni^2+^-NTA agarose beads, and following washing and elution, the bound proteins were identified by immunoblotting. While a protein of about 30 kDa recognized by the Strep-tag antibody seems to bind non-specifically to the beads, the full length CD1231-*Strep* as well as two probable degradation products, of about 37 and 40 kDa, were only detected in the presence of CD1234-His_6_ (S4B Fig). The two likely degradation fragments of CD1231-*Strep* may contain the CTD domain followed by the C-terminal extension (residues 405–505) consistent with the existence of a protease-sensitive site just downstream of the NTD in the recombinases from phages C31 and Bxb1 and from transposon TnpX [22, 38] (S4D Fig). These fragments may be retained by the Ni^2+^ column because they bind to the full-length CD1231-*Strep* recombinase or to CD1234-His_6_ (S4D Fig). In a different set of experiments, full-length CD1231-*Strep* was retained by the beads when these were pre-incubated with extracts prepared from BL21(DE3) cells producing CD1234-His_6_ and not Tgl-His_6_, an unrelated spore-associated protein from *B*. *subtilis* [39, 40], which accumulated to much higher levels than CD1234-His_6_ (S4C Fig). Together, these results indicate that CD1234 and CD1231 were part of a complex that formed in *E*. *coli* and suggest that CD1234 might control the activity of CD1231 by direct interaction.

### CD1231 and CD1234 are necessary and sufficient for *skin*^*Cd*^ excision in *E*. *coli*

**Fig 7:**
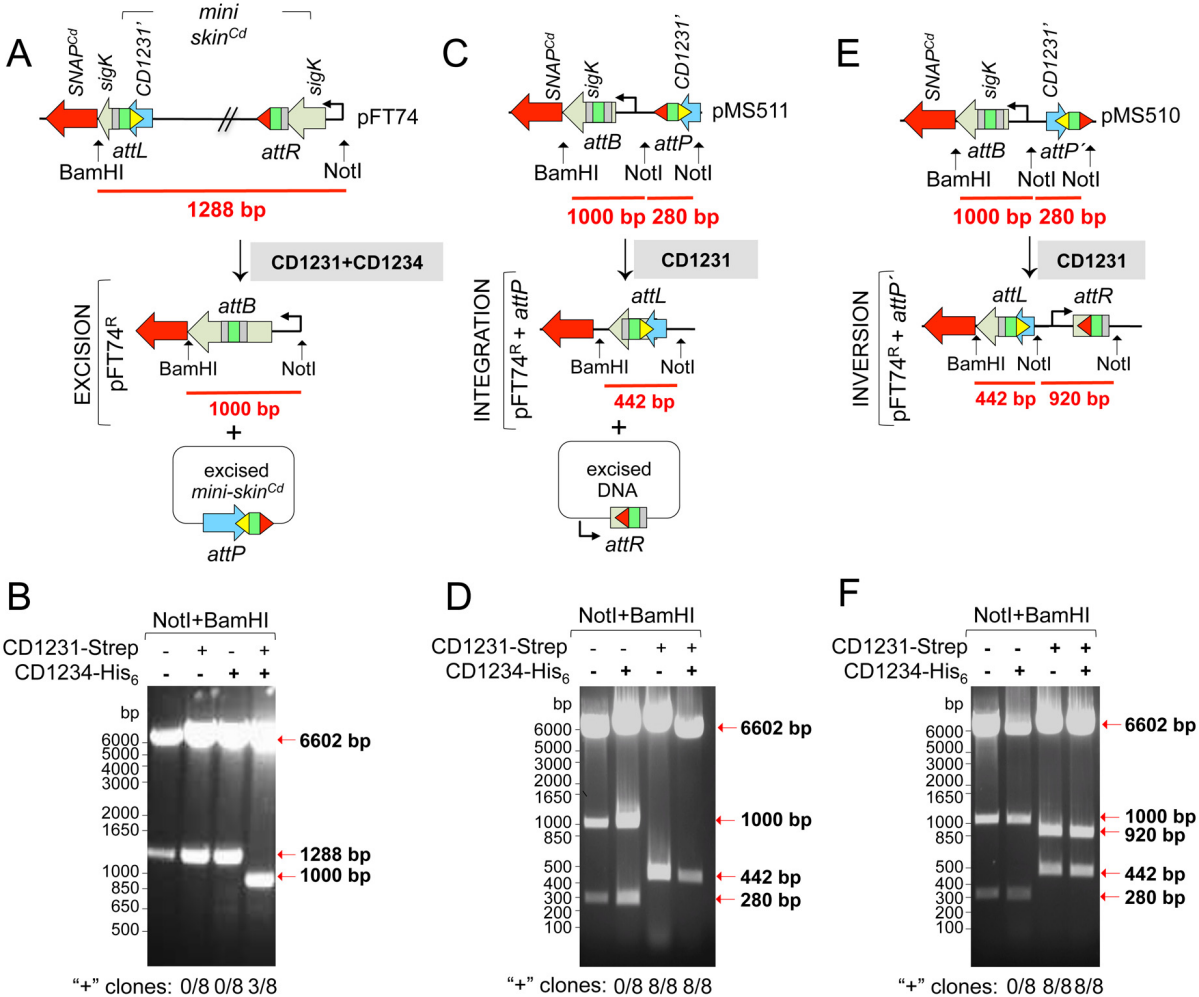
Detection of recombinase activity in *E*. *coli*. *E*. *coli* cells were transformed with plasmids pFT74 (**A**) or pFT74^R^-*attP* carrying a *skin*^*Cd*^-less *sigK* gene and *attP* in both possible orientations (**C** and **E**). The resulting strains were then transformed with plasmids containing the *CD1231-Strep*, *CD1234-His*_*6*_ or both genes (“+” signs) under the control of an IPTG inducible promoter. **A, C and E**: schematic representation of the recombination event (*skin*^*Cd*^ excision in A, *skin*^*Cd*^ integration in C and DNA inversion in E), the expected recombination products and the size of the intact and recombined inserts between the BamHI and NotI sites. *attP*´denotes an inverted *attP* site. **B, D and F:** analysis of the recombinant events following pFT74 or pFT74^R^-*attP* recovery from *E*. *coli* strains producing *CD1231-Strep*, *CD1234-His*_*6*_ or both genes (“+” signs) and digestion with BamHI and NotI. pFT74 and pFT74^R^+*attP* from strains that did not produce *CD1231-Strep* or *CD1234-His*_*6*_ were used as controls for non-recombined and recombined *sigK*, respectively. The size of molecular marker (in bp) is indicated on the left side of the panels. The “+” sign at the bottom of the panels indicate clones where the expected recombination event has occurred (8 clones were analyzed).

To test whether CD1234 and CD1231 were sufficient for *skin*^*Cd*^ excision, we used a heterologous host *E*. *coli*. The plasmid pFT74 carrying a mini *skin*^*Cd*^ element integrated into *sigK* was introduced in *E*. *coli* carrying plasmids for expression of *CD1231*, *CD1234* or the co-expression of both genes. The cells were first induced to produce CD1231, CD1234 or both. The plasmid pFT74 was then purified and examined for the recombination reaction by digestion with BamHI and NotI (Fig 7A). Digestion of the resulting recombined plasmid obtained after mini *skin*^*Cd*^ excision, termed pFT74^R^, with BamHI and NotI should produce a fragment of 1000 bp as compared to a fragment of 1288 bp for the parental plasmid carrying the mini-*skin* (pFT74). A fragment of 1000 bp was not isolated from cells producing neither CD1234 alone nor CD1231 alone or a mutant allele of CD1231 in which the putative catalytic serine in the NTD was changed to an alanine (S10A) (Figs 1B, 7B and S5). By contrast, pFT74^R^ was detected in cells in which both proteins were produced (Fig 7B). Together, these results show that CD1231 requires CD1234 as an auxiliary factor for the recombination reaction between *attL* and *attR* that results in *skin*^*Cd*^ excision.

### CD1231 is sufficient for *skin*^*Cd*^ integration in *E*. *coli*

While both CD1231 and CD1234 are required for *skin*^*Cd*^ excision in *E*. *coli*, we also wanted to test the involvement of these proteins in integration reactions. With that purpose, the *attP* site of the prophage-like element was inserted into pFT74^R^, which already contains the integration sequence *attB* as the result of the excision reaction (Fig 7A). Two plasmids were constructed, one with *attP* and *attB* in the same orientation (pMS511; Fig 7C) and one with *attB* and *attP* in opposite orientation (*attP*´ in pMS510; Fig 7E). These plasmids were introduced in *E*. *coli* cells carrying the plasmids for expression of *CD1231*, *CD1234* or both. Cells were induced to produce CD1231, CD1234 or both and then the plasmids were purified and examined for recombination events between *attP* and *attB* by digestion with BamHI and NotI. Two types of recombination events serve as readouts for the ability of CD1231 to catalyze DNA integration. A recombination event between *attP* and *attB* should result in the removal of the intervening DNA for pMS511 (Fig 7C) or in the inversion of the intervening DNA in the case of pMS510 (Fig 7E). We showed that CD1231 is necessary and sufficient for both types of recombination events involving *attP* and *attB* (Fig 7D and 7F). No recombined products of pMS510 or pMS511 were retrieved when the catalytically inactive CD1231 bearing the S10A substitution was produced, alone or together with CD1234 (S5 Fig).

These results showed that CD1231 is sufficient for the integration event that results from the recombination between *attP* and *attB*, but requires CD1234 for the excision event that results from the recombination reaction involving *attL* and *attR*. Thus, CD1234 is a recombination directionality factor (RDF) assisting CD1231 in *skin*^*Cd*^ excision.

### Deletion of the *skin*^Cd^ element causes premature activity of σ^K^

While the regulated excision of *skin*^*Cd*^ has been suggested as a critical mechanism for efficient sporulation in *C*. *difficile* [17, 27], a more recent study suggests that *skin*^*Cd*^ is not essential for the formation of heat-resistant spores in this organism [30]. However, *skin*^*Cd*^ excision controls the onset of σ^K^ activity. The deletion of the *skin* in a pro-less *sigK* strain in *B*. *subtilis* imposes changes in the mother cell line of gene expression leading to altered spore structure and functional properties, while the final titer of spores formed is reduced compared to the wild-type [41, 42]. To analyze more precisely the involvement of *skin*^*Cd*^ in sporulation, we took advantage of the 630Δ*erm*, which expressed *CD1234* under P_*tet*_ control, to obtain a congenic derivative of strain 630Δ*erm* lacking *skin*^*Cd*^ (summarized in S6A Fig). Addition of ATc during growth led to *skin*^*Cd*^ excision and after plating of the cells, DNA was extracted from isolated colonies. We identified several clones that carried a reconstructed *sigK* gene (S6B Fig, lane 1) but lacked the 5’ junction of *skin*^*Cd*^ in the chromosome (S6B Fig, lane 2) and the excised form of *skin*^*Cd*^ that was lost after cellular division (S6B Fig, lane 3). In a second step, a clone carrying an intact *sigK* gene was cured of the pDIA6103-P_tet_*CD1234* plasmid by successive cycles of growth and dilution in TY medium. After plating, Tm-sensitive clones that had lost pDIA6103-P_tet_*CD1234* were isolated. One clone was named 630Δ*erm* Δ*skin*^*Cd*^. Phase contrast microscopy experiments revealed the presence of free spores in both the 630Δ*erm* and 630Δ*erm* Δ*skin*^*Cd*^ strains (Fig 1C) and the titer of heat resistant spores measured 48 h and 72 h after inoculation in SM medium was almost identical for both strains (Fig 1C). Moreover, the percentage of sporulation measured for the two strains at 12 h (0.4% for 630Δ*erm* and 0.3% for 630Δ*erm* Δ*skin*), 18 h (1.6% and 1.1%) and 24 h (6.4% and 5.4%) following inoculation into SM also did not differ significantly. Thus, in agreement with the previous results using a *skin*^Cd^-less *sigK* gene expressed from a SpoIIID-independent promoter [30], deletion of *skin*^Cd^ did not appear to affect the final titer of spores and the kinetics of sporulation.

**Fig 8:**
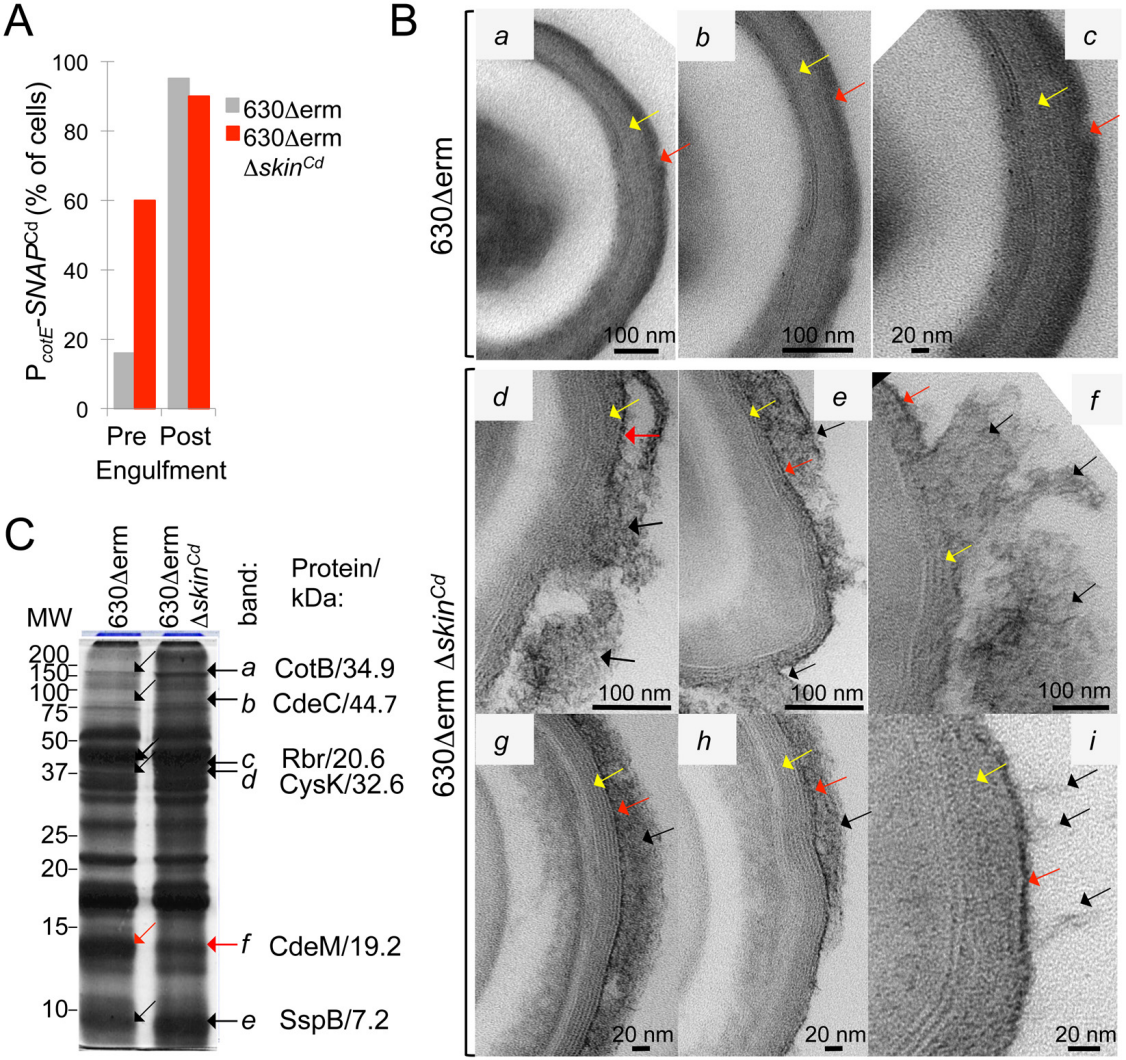
*skin*^Cd^ controls the time of σ^K^ activation and the fidelity of morphogenesis. **A:** quantitative analysis of the cells with fluorescence before and after the completion of engulfment in *C*. *difficile* cells carrying the P_*cotE*_-*SNAP*^*Cd*^ fusion (σ^K^-responsive) in strain 630Δ*erm* and 630Δ*erm Δskin*^*Cd*^. SNAP^Cd^ production was monitored as described in the legend for fig 3. **B**: purified spores were processed for imaging by transmission electron microscopy. Sections showing details of the spore surface layers for representative specimens are shown (the entire spores are shown in S7 Fig). The yellow arrow indicates the lamellar, inner structure of the coat, whereas the red arrow points to the more electrondense surface layer. The black arrows indicate material peeling off the surface of the spores. **C:** spores of the 630Δ*erm* and 630Δ*erm* Δ*skin*^*Cd*^ strains were purified by density gradient centrifugation, the spore surface proteins extracted and resolved by 15% SDS-PAGE. Bands *a* to *d* and *e* show increased representation in spores of the Δ*skin*^*Cd*^ strain, whereas band *f* shows reduced representation in Δ*skin*^*Cd*^ spores as compared to the 630Δ*erm*. The proteins identified in these bands by mass spectrometry are indicated, together with their predicted molecular weight.

We then analyzed transcription of *sigK* and of σ^K^ target genes by qRT-PCR in the 630Δ*erm* and 630Δ*erm* Δ*skin*^*Cd*^ strains. We first harvested the cultures between 10 h and 24 h of growth in SM. After RNA extraction, we tested the expression of *sigK* using oligonucleotides located on both sides of the *skin*^*Cd*^ insertion into *sigK* and of σ^K^ target genes. The results showed that the expression of *sigK* and of several *sigK* targets (*cotE*, *cotBC*, *sleC*, *cdeC*, *bclA1* and *bclA3*) was higher in the Δ*skin* strain than in the 630Δ*erm* strain (S2 Table). Lastly, we monitored the activity of σ^K^ at the single cell level using a P_*cotE*_-*SNAP*^*Cd*^ transcriptional fusion in the wild-type and Δ*skin*^*Cd*^ background. Fluorescence microscopy revealed that the signal intensity from the accumulation of TMR-Star-labeled SNAP^Cd^ did not differ significantly between wild-type and Δ*skin*^*Cd*^ sporangia, before or after engulfment completion. Strikingly, however, the Δ*skin*^*Cd*^ mutation increased the fraction of cells that showed P_*cotE*_-*SNAP*^*Cd*^ expression and hence σ^K^ activity, prior to engulfment completion, from 20% (WT) to 60% (Δ*skin*^*Cd*^) (Fig 8A). Thus, expression of a *skin*^*Cd*^-less *sigK* gene from its native promoter results in premature σ^K^ activity.

### Deletion of the *skin*^Cd^ element in strain 630Δ*erm* affects the assembly of the spore surface layers

Activation of σ^K^ in *B*. *subtilis* is tightly linked to engulfment completion, and mutations that cause its premature activation result in alterations in the properties of the resulting spores [41, 42]. σ^K^ plays an important role in the assembly of the spore coat [16, 17] and because σ^K^ was active prior to engulfment completion in a larger fraction of the 630Δ*erm* Δ*skin*^*Cd*^ sporangia as compared to the 630Δ*erm* strain, we examined ultrastructure and the polypeptide composition of the spore surface layers in the two strains. Spores were density gradient purified from cultures of the 630Δ*erm* and 630Δ*erm* Δ*skin*^*Cd*^ strains, and processed for electron microscopy. Under our conditions, spores of the 630Δ*erm* strain showed a more internal lamellar coat (Fig 8B, yellow arrows in panels *a* to *c*), covered by a more external electrondense layer (red arrows); this layer had a uniform and compact appearance around the entire spore (Figs 8B and S7; see also [17]). It may correspond to an exosporium-like layer, which in *C*. *difficile* seems to be closely apposed to the underlying coat ([3]; see also below). In contrast, the electrondense outer layer was less compacted in spores of the Δ*skin*^*Cd*^ strain, and absent in some sections (Fig 8B, black arrows in panels *d* to *i*). The disorganization of the outer layer presumably allowed the visualization of a thin electrondense layer forming the edge of the lamellar coat and in close apposition to it (Fig 8B, red arrows in panels *d* to *i*). However, this thin layer was missing in some sections and material from the inner lamellar layer appeared to peel off the spore in those sections (Fig 8B, black arrows in panels *d* and *f*). Overall, the lamellar coat layer also appeared to be less dense, making its structural organization more apparent (Fig 8B, *d* to *i* and S7 Fig). Spore surface proteins from both the coat and exosporium layers were extracted from spores of the 630Δ*erm* and Δ*skin*^*Cd*^ strains and resolved by SDS-PAGE. Several proteins showed increased extractability from spores of the Δ*skin*^*Cd*^ strain relative to the wild-type strain (Fig 8C, black arrows, bands *a* through *d* and *e*), whereas a protein of about 12 kDa (red arrow, band *f*) showed reduced extractability. Mass spectrometry analysis indicates that band *a* (size close to 200 kDa) contains CotB, whereas band *b* (size around 80 kDa) contains CdeC. Because the predicted sizes of CotB and CdeC are 34.9 kDa and 44.7 kDa, respectively, these two species may represent cross-linked products of these proteins. CotB and CdeC are likely critical determinants for the assembly of the spore exosporium [3]. Rubrerythrin (Rbr) with a predicted size of 20.6 kDa and CysK, with a predicted size of 32.6 kDa, are detected in bands *c* and *d*, at about 37 kDa (Fig 8C). At least Rbr, found in a band of about twice its predicted molecular weight, may form cross-linked homodimers, or be cross-linked to a protein of about 20 kDa. In contrast, a likely proteolytic fragment of the 19.2 kDa-exosporium protein CdeM, shows decreased representation or extractability in the mutant (Fig 8C, band *f*). The alterations in the assembly of CotB, CdeC and CdeM may explain at least in part the morphology of the outer spore layers in the Δ*skin*^*Cd*^ strain (Fig 8B); possibly, the Δ*skin* deletion affects mainly the assembly of the exosporium-like layer that in *C*. *difficile* seems to be juxtaposed to the coat, while the morphological alterations seem at the level of the coat may be in part a consequence of a misassembled exosporium-like layer. The increased representation of SspB (a forespore-specific protein) [18] may indicate that the spores of the mutant are more permeable or more sensitive to the extraction procedure (Fig 8C, band *e*). *In toto*, we conclude that deletion of *skin*^*Cd*^ affects the assembly of the *C*. *difficile* spore surface layers. The alterations in the assembly of the spore coat and of a more external possible exosporium-like layer are most likely due to the premature activity of σ^K^ in 630Δ*erm* Δ*skin*^*Cd*^ sporangia.

## Discussion

**Fig 9:**
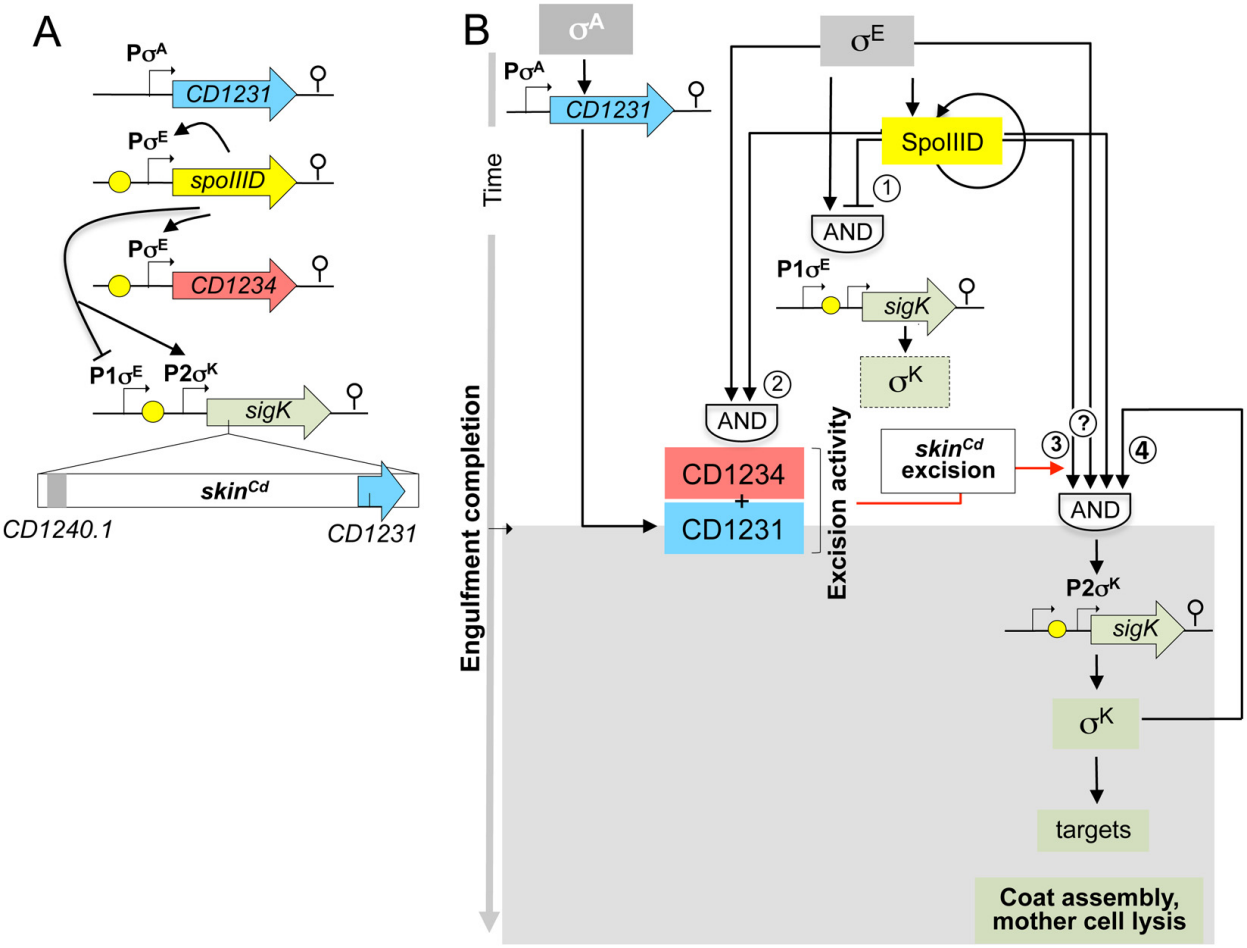
The sporulation network leading to *skin*^*Cd*^ excision and σ^K^ activation in *C*. *difficile*. **A:** schematic representation of the genes involved in *skin*^***Cd***^ excision and σ^K^ activation in *C*. *difficile*. Bent arrows show the promoters and yellow dots represent putative SpoIIID binding sites. The lines represent the control exerted by SpoIIID over the indicated genes. **B**: model of the organization of the mother cell transcriptional network. σ^E^ drives production of SpoIIID, which then activates transcription of both *CD1234* and *sigK* P2. *CD1234* and probably also *sigK* P2 (the later indicated by a question mark in A and B) are under the control of a coherent FFL with an AND gate logic (2 and 3 in the figure) established by σ^E^ and SpoIIID thought to delay *skin*^***Cd***^ excision (which also requires the CD1231 recombinase) and the main period of *sigK* transcription to post-engulfment sporangia. Then, a positive auto-regulatory loop combined with a positive control by SpoIIID leads to an increase in *sigK* transcription at P2 (4 in the figure) and to σ^K^ activity. SpoIIID may also repress transcription from the σ^E^-dependent *sigK* P1; the two regulatory proteins could thus form an incoherent FFL with an AND gate logic (1 in the figure), which could result in a low level of *sigK* transcription during engulfment. Transcription from *sigK* P1 would be shut-off coincidently with activation of P2. Possibly this would lead to a pulse in the production of σ^K^ in some cells in which *skin*^***Cd***^ is excised during engulfment (indicated as a dotted box in figure). As shown by correlating transcription of σ^K^-dependent genes with stages of morphogenesis using single cell analysis, the onset of the main period of σ^K^ activity coincides with the transition from phase grey to phase bright spores, presumably when synthesis of the spore cortex is finalized and the final stages in the assembly of the spore coat and exosporium begins.

The mother cell-specific excision of the *skin* element, in either *B*. *subtilis* or *C*. *difficile*, is essential for the production of a functional *sigK* gene. In *B*. *subtilis*, expression of the gene coding for SpoIVCA, the LSR responsible for *skin*^*Bs*^ excision is under the joint control of σ^E^ and SpoIIID [33]. Hence, *skin*^*Bs*^ excision is restricted to the terminal mother cell. Excision of the *skin*^*Cd*^ element is absolutely dependent on *CD1231* encoding a SpoIVCA homologue, but expression of this gene is not restricted to the mother cell. Rather, *CD1231* is transcribed from a σ^A^-dependent promoter and is expressed in vegetative cells and during sporulation in both compartments. An important finding of the present investigation is that excision of *skin*^*Cd*^ requires, in addition to CD1231, the product of a gene, *CD1234*, whose expression is under the control of both σ^E^ and SpoIIID (Fig 9). It is the requirement for CD1234 that restricts *skin*^*Cd*^ excision to the mother cell. However, the inactivation of CD1234 or SpoIIID does not reduce sporulation to the level observed for the *CD1231* or *sigK* gene disruption. CD1234 and SpoIIID thus appear less crucial for sporulation than σ^K^ or the catalytic function of the LSR, CD1231. It seems possible that CD1231 occasionally performs excision of *skin*^*Cd*^ without CD1234. Nevertheless, the normal requirement of CD1234 for *skin*^*Cd*^ excision in *C*. *difficile* is paralleled by its requirement for the CD1231-dependent excision of a mini-*skin*^*Cd*^ in a heterologous host, *E*. *coli* but conversely, CD1234 is not required for the CD1231-dependent *skin*^*Cd*^ integration. Thus, our recombination assays show that CD1234 is a RDF [22, 32, 37]. RDFs likely stimulate formation of the synapse complex between *attL* and *attR* by competing for inhibitory interactions involving the CC motifs of LSRs or inhibit formation or otherwise destabilize the synaptic complex formed between *attB* and *attP* possibly by stabilizing the auto-inhibitory activity of the CC motifs in this complex [22, 37]. Accordingly, the RDFs interact with the recombinase in solution, in line with our observation that CD1231 and CD1234 are part of a complex that formed in *E*. *coli*. Both structural studies and the isolation of mutations close or within the CC motif of the ϕC31 LSR that allows it to recombine *attL* and *attR* in the absence of the cognate RDF, suggest binding of the CC motifs by the RDF [22, 37]. More studies are needed to unravel the mechanism by which CD1234 cooperates with the CD1231 LSR to control *skin*^*Cd*^ excision. RDFs have been identified for several LSRs. However, they do not share significant sequence similarity and most of them appear to be small, basic proteins. In contrast, CD1234 is acidic like SprB, the RDF of phage SPβ [32]. SprB is required for the SprA recombinase-mediated excision of phage SPβ. A RDF assisting SpoIVCA in the excision of *skin*^*Bs*^ has not been identified, but the function of the *skin*^*Bs*^*-*encoded genes has not been inspected individually.

Recent studies have examined in detail the sporulation program of *C*. *difficile* and provided evidence that the temporal compartmentalization of sigma factor activity is less tightly regulated in *C*. *difficile* as compared to *B*. *subtilis* [16–18]. A difference between the sporulation programs in the two organisms may be the existence of a higher degree of redundancy in the control of gene expression at key stages in morphogenesis in *B*. *subtilis*. The control over *spoIVCA* and *sigK* transcription, *skin*^*Bs*^ excision, and proteolytical removal of the inhibitory pro-sequence ensures that σ^K^ is only active following engulfment completion in *B*. *subtilis* [41–43]. Since σ^K^ in *C*. *difficile* lacks a pro-sequence, the main period of σ^K^ activity may be mainly dictated by the time of CD1234 production and *skin*^*Cd*^ excision (Fig 9). In *B*. *subtilis*, both the *sigK* and *spoIVCA* genes are controlled by a coherent FFL involving σ^E^ and SpoIIID [19]. Thus, both the SpoIVCA-mediated *skin*^*Bs*^ excision and the transcription of *sigK* are delayed towards the end of the engulfment sequence, when the forespore signal that leads to pro-σ^K^ processing results in “just-in-time” activation of σ^K^. In *C*. *difficile*, transcription of *CD1234* and *sigK* decreased in a *spoIIID* mutant. We detected a common binding motif in the promoter region of these genes suggesting a direct effect of SpoIIID on their transcription. This motif is also present upstream of *spoIIID* itself allowing us to propose an auto-regulation for SpoIIID and to define a first consensus for the SpoIIID binding site of *C*. *difficile* (KRTAACARK) (S3A and S3B Fig) sharing partial similarity with the SpoIIID consensus of *B*. *subtilis* (S3C Fig) [19].

Our results show that expression of *CD1234* in *C*. *difficile* relies on a coherent FFL involving σ^E^ and SpoIIID (Fig 9B) [19]. Although transcription of *CD1234* is detected in the mother cell soon after asymmetric division and does not increase during or following engulfment completion, it is possible that the CD1234 protein only reaches a threshold level following engulfment completion. The effect of a *spoIIID* deletion on the transcription of *sigK* as assessed here using SNAP^Cd^ labeling and scoring of cells in which engulfment had been completed is less pronounced than previously reported on the basis of a more sensitive qRT-PCR analysis [18]. It seems possible that binding of SpoIIID to the *sigK* regulatory region represses the σ^E^-dependent P1 promoter towards the end of the engulfment process, while allowing activation of transcription from P2 (Fig 3A). If so, deletion of *spoIIID* would allow prolonged utilization of P1 by σ^E^, possibly explaining the reduced effect of the mutation following engulfment completion as seen here (Fig 3B). We do not presently know whether P2 is initially utilized by σ^E^ with the help of SpoIIID, and later by σ^K^. Further work is needed to test these possibilities. In any event, positive auto-regulation of *sigK* transcription at P2 may then increase production of active σ^K^ locking the cells in a late-mode of gene expression [16–18] (Fig 9B). This genetic architecture may allow delaying *sigK* transcription and *skin*^*Cd*^ excision to ensure that the main period of σ^K^ activity follows engulfment completion [17]. Thus, although the pro-sequence is absent from σ^K^, some level of redundancy also appears to be embedded in the activation of σ^K^ in *C*. *difficile*, with delayed transcription and rearrangement contributing to its timely activation. As we show here, the rearrangement is important, since expression of a *skin*^*Cd*^-less *sigK* gene increases σ^K^ activity during engulfment. In this regard, we comment on the possible function of *sigK* P1. If indeed this promoter is repressed through binding of SpoIIID to the downstream box, P1 would be subject to a type I incoherent FFL, which could result in a pulse of *sigK* transcription (Fig 9B) [44]. Provided *skin*^*Cd*^ excision also occurs, a pulse in *sigK* transcription may result in σ^K^ activation during engulfment in some cells in a population (Fig 9B). Given that a *skin*^*Cd*^-less *sigK* allele leads to alteration in the spore surface structure, we speculate that this regulatory scheme could produce spores with different structure and functional properties in a fraction of the population.

The presence of a *skin* element remains an exception in clostridia. Although the still more familiar designation of *C*. *difficile* was used herein, the organism differs significantly from typical Clostridial species, and was recently placed in the Peptostreptococcaceae family and renamed *Peptoclostridium difficile* [45]. Nevertheless, a prophage integrated into *sigK* is found in *Clostridium tetani* [46], in some strains of *C*. *botulinum* and in a strain of *C*. *perfringens* [47]. Absence of a *skin* element may be related to the involvement of σ^K^ in functions in addition to its role as a late mother cell-specific transcription factor. For instance, in *C*. *acetobutylicum* and *C*. *botulinum*, σ^K^ is required for entry into sporulation and for solventogenesis in the former organism and for cold and salt stress in the latter, while in *C*. *perfringens* σ^K^ is also involved in enterotoxin production [48]. It thus seems likely that *skin*^*Cd*^ together with the compartmentalized expression of *CD1234* helps preventing ectopic and heterochronic activity of σ^K^. This suggestion is in agreement with the observation that bypassing both the recombination and pro-protein processing levels of control leads to some activity of σ^K^ during stationary phase under conditions that do not support efficient sporulation by *B*. *subtilis* [42]. This inappropriate activity of σ^K^ is most likely the result of auto-regulation, and shows that even in *B*. *subtilis*, transcriptional control alone is not sufficient to restrict the activity of σ^K^ to the mother cell during late stages of spore development but also for proper sporulation as a pro-less *skin*-less strain of *B*. *subtilis* shows reduced sporulation [41, 42]. In contrast, the early activity of σ^K^ in the Δ*skin*^*Cd*^ strain of *C*. *difficile*, while causing alterations in coat assembly, does not affect the final titer of heat resistant spores. It is possible that the sporulation program in *C*. *difficile* is more permissive to changes in the proper timing of morphogenetic events [16–18]. It is also possible, however, that an additional as yet unidentified mechanism, controls the activity of σ^K^ in *C*. *difficile*.

Why the expression of CD1231 is not restricted to the mother cell is intriguing and might suggest the possible existence of other functions than *skin* excision for this recombinase. CD1231 may also function occasionally in the forespore or in vegetative cells, in the absence of CD1234, resulting in permanent elimination of *skin*^*Cd*^, as it is known that some strains of *C*. *difficile* including epidemic strains lack this element [27, 28, 49]. This possibility also raises the question of whether in those strains σ^K^ is recruited to additional functions, by analogy with *C*. *perfringens*, *C*. *botulinum* and *C*. *acetobutylicum*. Importantly, as we show that the premature activity of σ^K^ during sporulation in *C*. *difficile* imposes alterations to the assembly of the spore surface layers, we speculate that spores of the *skin*^*Cd*^-less epidemic strains may have alterations in structural and/or functional properties important for spore persistence and infection.

Reminiscent of the situation with *skin*, whose deletion alters assembly and function of the spore surface layers, it is noteworthy that the *B*. *subtilis* SPβ prophage is inserted into the *spsM* gene, required for the glycosylation of proteins at the spore surface, and that SPβ excision during sporulation depends on the mother cell-specific expression of the SPβ gene coding for the SprB RDF. Remarkably, SPβexcision during sporulation does not result in the assembly of phage particles, most likely because the prophage genes lack mother cell-specific promoters [32]. In different spore-formers, several other sporulation genes such as *spoVFB* encoding the dipicolinate synthase and *spoVR* involved in cortex formation are interrupted by phage-like elements that are able to excise during sporulation [32, 50]. Moreover, developmentally-controlled, recombinase-dependent excision of phage-like intervening elements also takes place in cyanobacteria where these elements, interrupting genes required for nitrogen fixation, are excised during heterocyst differentiation [51]. Insertion of phages or phage like elements in genes that are specifically expressed in terminal cell lines ensures their vertical transmission and minimizes cost for the host. However, regulated prophage excision may also occur in non-terminally differentiated cells. The *Listeria monocytogenes comK* gene, coding for the competence master regulatory protein, is interrupted by a prophage [52]. The DNA-uptake complex formed by the Com proteins is required for *L*. *monocytogenes* to escape from macrophage phagosomes and remarkably, excision of the *comK* intervening sequence is specifically induced during intracellular growth within phagosomes [52]. Also remarkably, prophage excision from *comK* as observed for SPβ excision in *B*. *subtilis* sporangia does not result in the production of phage particles [52]. Clearly, whether or not in terminally differentiated cells, host “domestication” of prophage excision, may lead to additional levels of genetic control over important cellular functions [22, 53].

## Materials and Methods

### Bacterial strains, growth and sporulation conditions

*C*. *difficile* strains and plasmids used in this study are presented in Table 3. *C*. *difficile* strains were grown anaerobically (5% H_2_, 5% CO_2_, and 90% N_2_) in Brain Heart Infusion (BHI, Difco), which was used for selection of conjugants, in TY (Bacto tryptone 30 g.L^-1^, yeast extract 20 g.L^-1^, pH 7.4) or in sporulation medium (SM) [54], which was used for sporulation assays. SM medium contained per liter: 90 g Bacto tryptone, 5 g Bacto peptone, 1 g (NH_4_)_2_SO_4_, 1.5 g Tris Base. When necessary, cefoxitin (Cfx; 25 μg/ml), thiamphenicol (Tm; 15 μg.ml^-1^) or erythromycin (Erm; 2.5 μg.ml^-1^) was added to *C*. *difficile* cultures. *E*. *coli* strains were grown in LB broth. When indicated, ampicillin (100 μg.ml^-1^) or chloramphenicol (15 μg.ml^-1^) was added to the culture medium. The antibiotic analog anhydrotetracycline (ATc; 100 ng.ml^-1^) was used for induction of the P_*tet*_ promoter present in derivatives of the *C*. *difficile* pDIA6103 vector [34].

**Table 3:** Bacterial strains used in this study.

Strain	Relevant Genotype/phenotype	Origin/Reference
***E*. *coli***		
Top10	F^-^ *mcr*A Δ (*mrr-hsd*RMS-*mcr*BC) f80*lac*ZΔM15 Δ*lac*X74 *deo*R, *rec*A1 *ara*D139 Δ (*ara-leu*)7697 *gal*K *rps*L(Str^R^) *end*A1 *nup*G	Invitrogen
HB101 (RP4)	*supE*44 *aa*14 *galK*2 *lacY*1 Δ(*gpt-proA*) 62 *rpsL*20 (Str^R^)*xyl-5 mtl-1 recA*13 Δ(*mcrC-mrr*) *hsdS*_B_(r_B_^-^m_B_) RP4 (Tra^+^ IncP Ap^R^ Km^R^ Tc^R^)	Laboratory stock
BL21(DE3)	*F*^*−*^*ompT gal dcm lon hsdS*_*B*_*(r*_*B*_^*-*^ *m*_*B*_^*-*^*)* λ*(DE3 [lacI lacUV5-T7 gene 1 ind1 sam7 nin5])*	Novagen
***C*. *difficile***		
630Δ*erm*	wild-type	Laboratory stock
AHCD532	630Δ*erm sigE*::*ermB*	[17]
AHCD535	630Δ*erm sigK*::*ermB*	[17]
AHCD601	630Δ*erm* pFT47- P_*sigK*_ -SNAP^*Cd*^	[17]
AHCD667	630Δ*erm* pFT47-P_*CD1231*_-SNAP^*Cd*^	This work
AHCD690	630Δ*erm spoIIID*::*ermB* pFT47- P_*sigK*_ -SNAP^*Cd*^	This work
AHCD695	630Δ*erm* pFT47- P_*cotE*_*-* SNAP^*Cd*^	[17]
AHCD699	630Δ*erm sigK*::*ermB* pFT47- P_*cotE*_*-* SNAP^*Cd*^	[17]
AHCD700	630Δ*erm spoIIID*::*ermB* pFT47- P_*cotE*_*-* SNAP^*Cd*^	This work
AHCD758	630Δ*erm CD1234*::*ermB* pFT47- P_*cotE*_*-* SNAP^*Cd*^	This work
AHCD763	630Δ*erm CD1231*::*ermB* pFT47- P_*cotE*_*-* SNAP^*Cd*^	This work
AHCD787	630Δ*erm* pFT74 (SigK-SNAP, *skin*^+^)	This work
AHCD800	630Δ*erm spoIIID*::*ermB* pFT74 (SigK-SNAP, *skin*^+^)	This work
AHCD851	630Δ*erm* Δ*skin*^*Cd*^ pFT47- P_*cotE*_*-*SNAP^*Cd*^	This work
CDIP224	630Δ*erm spoIIID*::*ermB*	[18]
CDIP345	630Δ*erm sigE*::*ermB* pFT47-P_*CD1234*_-SNAP^*Cd*^	This work
CDIP389	630Δ*erm* pFT47-P_*CD1234*_-SNAP^*Cd*^	This work
CDIP396	630Δ*erm CD1234*::*ermB*	This work
CDIP397	630Δ*erm CD1234*::*ermB* pMTL84121-*CD1234*	This work
CDIP399	630Δ*erm spoIIID*::*ermB* pFT47-P_*CD1234*_-SNAP^*Cd*^	This work
CDIP526	630Δ*erm CD1231*::*ermB*	This work
CDIP533	630Δ*erm CD1231*::*ermB* pMTL84121-*CD1231*	This work
CDIP560	630Δ*erm* pDIA6103	This work
CDIP561	630Δ*erm* pDIA6103-*CD1234*	This work
CDIP562	630Δ*erm spoIIID*::*ermB* pDIA6103	This work
CDIP563	630Δ*erm spoIIID*::*ermB* pDIA6103-*CD1234*	This work
CDIP564	630Δ*erm sigE*::*ermB* pDIA6103	This work
CDIP565	630Δ*erm sigE*::*ermB* pDIA6103-*CD1234*	This work
CDIP566	630Δ*erm CD1231*::*ermB* pDIA6103	This work
CDIP567	630Δ*erm CD1231*::*ermB* pDIA6103-*CD1234*	This work
CDIP568	630Δ*erm CD1234*::*ermB* pDIA6103	This work
CDIP569	630Δ*erm CD1234*::*ermB* pDIA6103-*CD1234*	This work
CDIP583	630Δ*erm* Δ*skin*^*Cd*^	This work
**Plasmids**		
pMTL007	ClosTron plasmid; *catP*; intron containing *ermB*::RAM (Cm^R^/Tm^R^)^1^	[55]
pMTL84121	*Clostridia* modular plasmid; *catP* (Cm^R^/Tm^R^)	[56]
pDIA6103	pRPF185 Δ*gusA*	[34]
pFT38	pMTL84121-*sigK*^*skin+*^ (Cm^R^/Tm^R^)	[17]
pFT47	pMTL84121-*SNAP*^*Cd*^ (Cm^R^/Tm^R^)	[17]
pFT51	pFT47 P_*sigK*_-*SNAP*^*Cd*^	[17]
pFT69	pFT47 P_*cotE*_-SNAP^*Cd*^	[17]
pFT74	pMTL84121 P-*sigK5’-*^*skin+*^-SigK-SNAP^*Cd*^	This work
pMS469	pFT47 P_*CD1231*_-SNAP^*Cd*^	This work
pMS484	pET33b-*CD1234-His*_*6*_	This work
pMS486	pET16b-*CD1231-Strep tag*	This work
pMS494	pETDuet-1- *CD1234-His*_*6*_	This work
pMS499	pETDuet-1- *CD1231-Strep tag*	This work
pMS500	pETDuet-1- *CD1234-His*_*6*_*- CD1231-Strep tag*	This work
pMS510	pMTL84121-*sigK*-SNAP^*Cd*^-*attP’*	This work
pMS511	pMTL84121-*sigK*-SNAP^*Cd*^-*attP*	This work
pMS512	pETDuet-1- *CD1231*^*S10A*^*-Strep tag*	This work
pMS513	pETDuet-1- *CD1234-His*_*6*_*- CD1231*^*S10A*^*-Strep tag*	This work
pDIA6314	pMTL007::Cdi-*CD1234*-89a	This work
pDIA6346	pMTL007::Cdi-*CD1231*-206a	This work
pDIA6192	pFT47-P_*CD1234*_-SNAP^*Cd*^	This work
pDIA6307	pMTL84121-*CD1234*	This work
pDIA6348	pMTL84121-*CD1231*	This work
pDIA6353	pDIA6103-*CD1234*	This work
pDIA6382	pGEMT-*attP*	This work
pLOM4	pET30a(+)-*tgl-His*_6_	[40]

Sporulation assays were performed as follows. After 12 h, 18 h, 24 h, 48 h or 72 h of growth in SM medium, 1 ml of culture was divided into two samples. To determine the total number of cells, the first sample was serially diluted and plated on BHI with 0.1% taurocholate (Sigma-Aldrich) to ensure efficient spore germination [5]. To determine the number of spores, the vegetative bacteria of the second sample were heat killed by incubation for 30 min at 65°C prior to plating on BHI with 0.1% taurocholate. The rate of sporulation was determined as the ratio between the number of spores/ml and the total number of bacteria/ml (x100).

### Construction of *C*. *difficile* strains

The ClosTron gene knockout system [55] was used to inactivate the *CD1231* and *CD1234* genes in the strain 630Δ*erm*, to produce strains CDIP526 (*CD1231*::*erm*) and CDIP396 (*CD1234*::*erm*) (Table 3). Primers to retarget the group II intron of pMTL007 to these genes (S1 Table) were designed using the Targetron design software (http://www.sigmaaldrich.com). The PCR primer sets were used with the EBS universal primer and intron template DNA to generate, by overlap extension PCR, a 353-bp product that would facilitate intron retargeting to *CD1231* or *CD1234*. The PCR products were cloned between the HindIII and BsrGI sites of pMTL007 to yield pDIA6346 (pMTL007::Cdi-*CD1231-*206a) and pDIA6314 (pMTL007::Cdi-*CD1234*-89a). pDIA6346 and pDIA6314 were introduced into *E*. *coli* HB101 (RP4) and the resulting strains subsequently mated with *C*. *difficile* 630Δ*erm*. *C*. *difficile* transconjugants were selected on BHI agar containing Tm and Cfx and then plated on BHI agar containing Erm. Chromosomal DNA of transconjugants was purified using the Instagene kit (Biorad). The Erm resistance phenotype was due to the splicing of the group I intron from the group II intron following integration, as shown by PCR using the ErmRAM primers (ErmF and ErmR). To verify integration of the Ll.LtrB intron into the right gene targets, we used PCR with two primers flanking the insertion site in *CD1231* (IMV736/IMV737) or *CD1234* (IMV695/IMV696), the intron primer EBSu and the *CD1231-* (IMV736) or *CD1234-*specific primers (IMV696) (S2 Fig). The Southern blot probe was generated by PCR using pMTL007 as a template and the primer pair OBD522 and OBD523 (S1 Table), yielding a 374 bp PCR product that hybridized with the group II intron. Southern blot analyses were performed as previously described [17, 18].

For complementation studies, the *CD1231* gene with its promoter (positions -192 to +1569 from the translational start site) and the *CD1234* gene with its promoter (-168 to +343 from the translational start site) were amplified by PCR using primers IMV728 and IMV729 or IMV695 and IMV696, respectively (S1 Table). The PCR fragments were cloned between the XhoI and BamHI sites of pMTL84121 [56] to produce pDIA6307 (*CD1234*) and pDIA6348 (*CD1231*). These plasmids were introduced into *E*. *coli* HB101 (RP4) and then transferred by conjugation into CDIP526 (*CD1231*::*erm*) to produce CDIP533, and into CDIP396 (*CD1234*::*erm*) to produce CDIP397 (Table 3).

### Transcriptional *SNAP*^*Cd*^ fusions

To construct transcriptional *SNAP*^*Cd*^ fusions to the *CD1231* and *CD1234* promoters, 485 and 139 bp DNA fragments containing the promoter region of each gene were PCR-amplified using genomic DNA from strain 630Δ*erm* and primer pairs CDsigK3’ Fw/CDsigK3’_EcoRI Rev or IMV677/IMV678, respectively. These fragments were cloned into pFT47 [17] to create pMS469 and pDIA6192 (Table 3). Plasmid pDIA6192 was transferred to 630Δ*erm*, 630Δ*erm spoIIID*::*erm* or 630Δ*erm sigE*::*erm* and pMS469 was transferred to 630Δ*erm* by conjugation from derivatives of *E*. *coli* HB101 (RP4) (Table 3).

### Construction of a translational reporter for *skin*^*Cd*^ excision

Primers PCDsigK Fw and CDsigK5’Rev (S1 Table) were used to amplify a fragment from the *sigK* gene using pFT38 [17] as the template. The resulting 819 bp fragment encompasses 415 bp of the *sigK* regulatory region and the 5´-end of the interrupted coding region, 404 bp downstream of the *sigK* translational start site [18]. This fragment was cleaved with NotI and EcoRI. In a second PCR, the region containing the 3´-end of the coding sequence of *sigK*, and 180 bp upstream of this position, was PCR amplified using primers RecFP-Fw and SigK-SNAP Rev, yielding a 445 bp fragment. This fragment was cleaved with EcoRI and BamHI. The two fragments were inserted between the NotI and BamHI sites of pFT58 to produce pFT74 (Fig 4A). pFT74 was transferred by conjugation into *C*. *difficile* 630Δ*erm* and 630Δ*erm spoIIID*::*erm* (Table 3).

### Construction of *CD1234* inducible strains

To express the *CD1234* gene under the control of a P_*tet*_ inducible promoter, the *CD1234* gene with its ribosome-binding site (positions -19 to + 343 from the translational start codon) was amplified using primers IMV720 and IMV696 (S1 Table). The resulting PCR product was digested with StuI and BamHI and cloned into pDIA6103, a derivative of pRPF185 lacking the *gusA* gene [34]. Using the *E*. *coli* HB101 (RP4) strain containing either pDIA6103 or pDIA6353 (pDIA6103-*CD1234*), these plasmids were transferred by conjugation into *C*. *difficile* 630Δ*erm*, 630Δ*erm spoIIID*::*erm*, 630Δ*erm sigE*::*erm*, 630Δ*erm CD1234*::*erm* or 630Δ*erm CD1231*::*erm*.

### Detection of *skin*^*Cd*^ excision by PCR or qPCR

The various strains of *C*. *difficile* containing either pDIA6103 or pDIA6103-*CD1234* (Table 3) were grown overnight in TY containing 0.025% of taurocholate. The pre-cultures were diluted 100-fold in TY medium and the resulting cultures incubated at 37°C for 4 h. To induce expression of *CD1234*, ATc (100 ng/ml) was then added to the medium. After 2 h of incubation, the cells were harvested by centrifugation and the chromosomal DNA was extracted from each strain using the Puregene Yeast/Bact kit (QIAGEN). To quantify the reconstruction of *sigK* associated to *skin*^*Cd*^ excision, quantitative PCR (qPCR) was performed using primers upstream (QRTBD325) and downstream (QRTBD326) of the *skin*^*Cd*^ insertion site. A qPCR of the DNA-PolIII gene was used as a control. For each strain, a dilution of the culture was also plated on BHI. Eight clones per strain were re-isolated on BHI plates, and DNA was extracted from each using the Instagene kit (Biorad). To detect the presence of *skin*^*Cd*^ into the chromosome, the 5’ junction of the *skin*^*Cd*^ element was PCR-amplified using primers OBD0742 (in *CD1231*) and QRTBD326 (in *sigK*).

### Construction of a Δ*skin*^*Cd*^ mutant

A derivative of strain 630Δ*erm* lacking the *skin*^*Cd*^ was obtained as follows. Strain 630Δ*erm* (pDIA6103-*CD1234*) was grown 4 h in TY. After inducing expression of *CD1234* expression, the cells were serially diluted, plated on BHI and DNA extracted for several clones. To verify *skin*^*Cd*^ excision, PCR was performed with primers flanking the site of *skin*^*Cd*^ insertion (QRTBD326 and IMV833) or the 5’ junction of the *skin*^*Cd*^ element (OBD0742 in *CD1231* and QRTBD326 in *sigK*). One clone in which *sigK* was amplified and lacking the 5’ junction of *skin*^*Cd*^ *skin* was selected. To cure the selected clone of pDIA6103-*CD1234*, cells were diluted 6-fold in TY medium before plating on BHI. About 70% of the clones obtained after plating were Tm^S^ and had lost pDIA6103-*CD1234* as checked by PCR.

### RNA extraction and quantitative RT-PCR

Total RNA was isolated from strains 630Δ*erm* and 630Δ*erm* Δ*skin*, from the *CD1231* and *CD1234* mutants and from the complementation strains. These strains were grown in SM medium, the cells were collected by centrifugation, resuspended in RNApro^™^ solution and RNA extracted using the FastRNA Pro Blue Kit, according to the manufacturer’s instructions (MP Biomedicals). Quantitative real-time PCR (qRT-PCR) analysis was performed as previously described [57]. The primers used for each marker are listed in S1 Table. In each sample, the quantity of cDNAs of a gene was normalized to the quantity of cDNAs of the DNApolIII gene. The relative change in gene expression was recorded as the ratio of normalized target concentrations (ΔΔCt) [58].

### Assay for recombinase activity and identification of *attP*

The *CD1234* coding region was PCR-amplified using primers *CD1234-*Fw and -Rev. The resulting 252 bp DNA fragment was cleaved with NdeI and XhoI and cloned between the same sites of pET33b (Novagen), creating pMS484. *CD1234* fused to the *His*_*6*_*-tag* was PCR-amplified from pMS484 with primers CD1234 Fw and T7ter, cleaved with NdeI and cloned between the NdeI and EcoRV sites of pETDuet-1, to create pMS494. The *CD1231* coding region was amplified using primers CD1231-Fw and -Rev. The resulting 1518 bp fragment was digested with NcoI and SalI and ligated to pFN127 (a pET16b derivative carrying a *Strep-tag*) to yield pMS486. *CD1231* fused to the *Strep-tag* was removed from pMS486 by digestion with NcoI and XhoI and cloned into pETDuet-1 producing pMS499, or in pMS494 to give pMS500. We used pMS499 and pMS500 (see above) and *CD1231*-specific primers to convert the serine codon at position 10 to an alanine codon producing pMS512 and pMS513, respectively. Derivatives of BL21(DE3) were constructed bearing pFT74 and pMS494 and either pMS499, pMS500, pMS512, or pMS513. The resulting strains were grown to an OD_600nm_ of about 0.6, induced with 1 mM isopropyl-D-thiogalactopyranoside (IPTG), and incubated for 3 h before the cells were harvested. Plasmid DNA was extracted and analyzed by cleavage with NotI and BamHI. pFT74^R^ results from mini*skin*^*Cd*^ excision, by recombination, from pFT74.

The *attP* site was obtained by PCR using IMV825 and IMV824 and DNA extracted from 630Δ*erm* after 24 h of growth in SM. The PCR product was first cloned into pGEM-T-easy (Promega) and the *attP* site was then released with NotI and inserted into pFT74^R^, in both directions, to produced pMS510 and pMS511. Derivatives of BL21(DE3) were constructed, bearing pMS510 or pMS511 and either pMS494, pMS499, pMS500, pMS512, or pMS513. The various strains were grown to an OD_600nm_ of about 0.6, induced with 1 mM IPTG, and incubated for 3 h before the cells were harvested. Plasmid DNA was extracted and cleaved with BamHI and NotI for the detection of recombination events.

### Pull-down assays

Derivatives of BL21 (DE3) bearing plasmids for the production of CD1231-Strep, CD1234-His_6_ or Tgl-His_6_, were grown to mid-log phase (OD_600nm_≈0.6) in LB, and induced with 1 mM IPTG for 3 hours before the cells were harvested by centrifugation. The cell sediment was resuspended in 1-ml portions of buffer A [100 mM NaCl, 10 mM Tris-HCl (pH 8.0), 10% glycerol] per 50 ml of induced culture and lysed in a French pressure cell (18,000 lb/in^2^). In one set of assays, the lysates containing CD1231-Strep, CD1234-His_6_, or both, were cleared by centrifugation and 1 ml of each lysate was independently incubated with 50 μl of a 50% slurry of Ni^2+^-NTA agarose (Qiagen) at room temperature for 30 min. The Ni^2+^-NTA agarose was washed three times in buffer B (same as A but with 200 mM NaCl). In another set of assays, lysates containing either CD1234-His_6_ or Tgl-His_6_ were incubated with the Ni^2+^-NTA beads as above, and then incubated with a CD1231-Strep-containing extract for a further 30 min at room temperature. The beads were washed as described above. In all cases, the washed beads with bound proteins were resuspended in a final volume of 30 μl. The samples were resolved on 15% SDS-PAGE and subject to immunoblotting. Anti-His_6_ or anti-Strep antibodies were used at a 1:1000 dilution, and a rabbit secondary antibody conjugated to horseradish peroxidase (from Sigma) was used at a 1:10000 dilution. The immunoblots were developed with enhanced chemiluminescence reagents (Amersham Pharmacia Biotech).

### Spore production, purification and spore coat extraction

For spore production, 5 ml of BHI was inoculated with of *C*. *difficile* and grown for 12 h at 37°C in anaerobic conditions. Fresh BHI (125 ml) was then inoculated with 1.25 ml of the pre-inoculum and the cultures incubated at 37°C under anaerobic conditions for 7 days. Cells were collected by centrifugation (for 10 min at 4800xg), resuspended in cold water and stored overnight at 4°C. Spores were then purified with a 42% Gastrografin (Schering) step gradient, as previously described [59]. The pellet was washed 10 times with cold water, and stored at -20°C. To analyze the profile of spore coat proteins, the spores were resuspended in extraction buffer (0.125 mM Tris-HCl, 5% β-mercaptoetanol, 2% SDS, 0.025% bromophenol blue, 0.5 mM DTT, 5% glycerol, pH 6.8) to a final OD_580_ of 4, and boiled. The extracted proteins were resolved by 15% SDS-PAGE and visualized by Coomassie brilliant blue R-250 staining. For identification, protein bands were excised and digested with trypsin, before analysis by matrix-assisted laser desorption ionization mass spectrometry.

### Microscopy and image analysis

Samples (1 ml) were withdrawn from SM cultures at the desired times following inoculation, and the cells collected by centrifugation (4000 x*g*, for 10 min, at 4°C). The cells were washed with 1ml of PBS and resuspended in 0.1 ml of PBS supplemented with the membrane dye Mitotracker Green (MTG) at 0.5 μg.ml^-1^ and the DNA stain DAPI (4',6-diamidino-2-phenylindole; 50 μg.ml^-1^) (Invitrogen). For SNAP staining, culture samples of 1 ml were stained for 30 min with 250 nM SNAP-Cell TMR-Star (New England Biolabs) as described before [17]. Cells were washed four times by centrifugation (4000 *g*, 5 min) and ressupended in 1ml of PBS. The cells were resuspended in 1 ml of PBS containing 0.5 μg.ml^-1^ of MTG. Cells were mounted on 1.7% agarose coated glass slides and imaged as previously described [60]. Images were analyzed using the Metamorph software suite version 5.8 (Universal Imaging). For quantification of the SNAP^Cd^-TMR Star signal, 6x6 pixel regions were defined in the desired cell and the average pixel intensity was calculated, and corrected by subtracting the average pixel intensity of the background. Small fluctuations of fluorescence among different fields were corrected by normalizing to the average pixel intensity obtained for the intrinsic autofluorescence of *C*. *difficile* cells [61].

### Transmission Electron Microscopy

For thin sectioning transmission electron microscopy (TEM) analysis, *C*. *difficile* were purified by density gradient centrifugation as described above. Samples were processed for TEM as described previously [62].

## Supporting Information

S1 Fig Sequence alignment of four serine integrases, SpoIVCA from *B*. *subtilis*, CD1231 from *C*. *difficile*, and the recombinases from the C31and A118 phages. The alignment was generated using T-coffee (www.tcoffee.org). The color code indicates conservation, as indicated by the scale on the lower right corner. The conserved motifs are indicated, as well as the boundaries between the N- (NTD) and C-terminal (CTD) domains of the recombinase. The black dot indicates the catalytic nucleophile (Ser10) and the brown dots additional catalytic residues in CD1231. The position homologous to residue Glu449 in the ϕC31 recombinase is indicated by a red triangle. The E449K substitution in the ϕ C31 protein results in a “hyperactive” recombinase able to recombine *attL* and *attR* in the absence of the RDF.
(TIF)

S2 Fig Inactivation of the *CD1231* or *CD1234* gene in *C*. *difficile* using the ClosTron system. **A**: schematic representation of gene inactivation by a type II Intron with an associated Retro-transposon-Activated Marker (RAM) [54]. The group II intron (bracket), originally in pMTL007 (top), carries a RAM element interrupting an ermB determinant (white). The intron was retargeted to *CD1231* or *CD1234* (blue) by altering the IBS, EBS1 and EBS2 sequences (grey and white stripes; top) by overlapping PCR. Splicing out of the td group I intron from the *ermB* gene in the RAM restores a functional marker allowing positive selection of mutants following intron integration. Primers used to confirm the integration and orientation of the type II intron are also indicated (bottom). **B**: chromosomal DNA of Em^R^ *C*. *difficile* conjugants obtained during CD1231 inactivation by Clostron and of strain 630Δ*erm* were screened by PCR using primer pairs RAM-F/R to confirm splicing out of the group I intron in the mutant (lane 1 and 2). We also performed PCR using chromosomal DNA of strain 630Δ*erm* and of the *CD1231* mutant (lane 3 and 4) with the intron primer EBSu and with a primer in *CD1231* (IMV737). To verify the integration of the Ll.LtrB intron into the right gene targets, we further performed PCR using chromosomal DNA of strain 630Δ*erm* and of the *CD1231* mutant (lane 5 and 6) with two primers flanking the insertion site in *CD1231* (IMV737-IMV736). Chromosomal DNA from the 630Δ*erm* strain corresponded to lane 2, 3 and 6 while chromosomal DNA of the *CD1231* mutant corresponded to lane 1, 3 and 5. The smart ladder (Eurogentec) was used as a molecular weight marker. **C**: chromosomal DNA of Em^R^ *C*. *difficile* conjugants obtained during CD1234 inactivation by Clostron and of strain 630Δ*erm* were screened by PCR using primer pairs RAM-F/R to confirm splicing out of the group I intron in the mutant (lane 1 and 2). To verify the integration of the Ll.LtrB intron into the right gene targets, we further performed PCR using chromosomal DNA of strain 630Δ*erm* and of the *CD1234* mutant with the intron primer EBSu and with a primer in *CD1234* (IMV696) (lane 3 and 4) or with two primers flanking the insertion site in *CD1234* (IMV695-IMV696) (lane 5 and 6). Chromosomal DNA from the 630Δ*erm* strain corresponded to lane 2, 4 and 6 while chromosomal DNA of each mutant corresponded to lane 1, 3 and 5. The smart ladder (Eurogentec) was used as a molecular weight marker. **D**: southern blot analysis of genomic DNA from *C*. *difficile* 630Δ*erm*, CDIP526 (*CD1231*::*erm*) and CDIP396 (*CD1234*::*erm*) mutant strains with an intron probe. Chromosomal DNA (6 μg in each reaction) was digested with *Hind*III. Southern blot analyses were performed using Amersham ECL Direct Nucleic Acid labelling and detection reagents, in accordance with the manufacturer's guidelines and visualised using Super Signal West Femto Maximum Sensitivity Substrate (Thermo Scientific). The probe was produced by PCR using OBD522 and OBD523 primers (S1 Table), designed within the group II intron sequence.
(TIF)

S3 Fig The promoter region of the *C*. *difficile spoIIID* gene (A) and the SpoIIID box (B and C). Panel A: The DNA sequence immediately uptream of the coding region of *spoIIID* is presented. The transcriptional start site (+1, red) as previously mapped [34], the -10 and -35 promoter elements (green) that match the consensus for σ^E^ recognition (represented below the sequence), SpoIIID boxes (yellow), and the start codon of *spoIIID*, are indicated. Panel B corresponds to an alignment of the SpoIIID box identified upstream of *CD1234*, *sigK* and *spoIIID*. The sequence logo was created from on the WebLogo website (http://weblogo.berkeley.edu) using SpoIIID boxes identified in this study. The asterisk means that the sequence of the SpoIIID2 box is in opposite orientation. Panel C shows the SpoIIID box of *B*. *subtilis* as described in Eichenberger *et al* [19].
(TIF)

S4 Fig CD1231 and CD1234 directly interact. **A**: whole cell extracts were made from *E*. *coli* BL21(DE3) derivatives induced with IPTG to produce CD1231-*Strep* (expected size 59 kDa), CD1234-His_6_ (15 kDa), or both (as indicated by the «+ » signs). **B**: the proteins were detected in the extracts by immunoblotting (first three lanes) using anti-*Strep* (top) or anti-His_6_ antibodies (bottom). CD1231-*Strep* was detected as a species migrating around 60 kDa as indicated (the asterisks represent likely degradation products). CD1234-His_6_ was detected as a species of about 12 kDa, as indicated. The last three lanes of panel B represent the results of a pull down assay, in which CD1231-*Strep* (lane 4), CD1234-His_6_ (lane 5) or the two co-produced proteins (lane 6) were incubated with Ni^2+^-NTA agarose beads. CD1234-His_6_ alone (lane 5) binds to the beads. Residual binding of CD1231-Strep to the beads (lane 4) was also detected, but retention of the protein was greatly increased in the presence of CD1234-His_6_ (lane 6). **C**: the left side of the panel shows the immnoblot analysis of whole cell extracts prepared from *E*. *coli* BL21(DE3) strains producing the indicated proteins (« + » signs) individually. The extracts containing Tgl-His_6_ [39,40] or CD1234-His_6_ were incubated with the Ni^2+^-NTA agarose beads after which the extract containing CD1231-Strep was added. Proteins were eluted, resolved by SDS-PAGE and subject to immunoblot analysis with anti-*Strep* (top) or anti-His_6_ antibodies (bottom). The asterisks represent likely degradation products. **D**: diagram of the domain organization of CD1231 (as in Fig 1B) showing the location of a protease-sensitive site at the end of the NTD, in the recombinases from phages C31 and Bxb1 and from transposon TnpX. The calculated size of the full-lenght protein and of two fragments containing the C-terminal end of the protein (CTD and C-terminal extension) is shown. Fragments of about 37 and 40 kDa most likely containing the C-terminal end of CD1231-*Strep* may interact with CD1234-His_6_ (see main text for details).
(TIF)

S5 Fig The CD1231^S10A^ single amino acid substitution abolishes recombinase activity. *E*. *coli* cells were transformed with plasmids pFT74 and pMS510 and pMS511 corresponding to pFT74^R^+*attP* [carrying a recombined *sigK* gene and containing the *attP* site in both possible orientations (*attP* or *attP*´; see also Fig 7)]. Each *E*. *coli* strain containing one of these plasmids was then transformed with plasmids expressing either CD1231-*Strep* or CD1231^S10A^-*Strep*, and *CD1234-His*_*6*_ or both under the control of an IPTG inducible promoter. Plasmids were recovered from *E*. *coli* strains and analyzed by digestion with NotI and BamHI. Plasmid DNA from pFT74 and pFT74^R^+*attP* were used as controls for non-recombined and recombined *sigK*, respectively. The size of molecular size marker (in bp) is indicated on the left side of the panels. The “+” sign identifies clones where recombination has occurred (a total of 8 clones were analyzed).
(TIF)

S6 Fig Construction and analysis of a 630Δ*erm* Δ*skin*^*Cd*^ mutant. **A:** strategy of construction of the 630Δ*erm* Δ*skin*^*Cd*^ strain. A plasmid (pDIA6103-*CD1234*) carrying the gene *CD1234* expressed under the control of a P_*tet*_ promoter was transferred into strain 630Δ*erm* by conjugation. After 4 h of growth, *CD1234* expression was induced with ATc for 2 h and cells were plated on BHI. The loss of the plasmid was obtained and verified as outlined in the Materials and Methods section. **B:** verification of the *skin*^*Cd*^ excision into the chromosome of the strain 630Δ*erm* Δ*skin*^*Cd*^ after 5 h of growth in TY medium (vegetative cells). Lane 1: PCR recombined *sigK* (qRTBD326-IMV833); lane 2: PCR *skin*^*Cd*^ junction (OBD742-qRTBD326); lane 3: PCR excised form (IMV824-IMV825). **C:** detection of *skin*^*Cd*^ in strain 630Δ*erm* during vegetative growth in TY medium (4 h). The gel shows the PCR products obtained with the following primer pairs: lane 1, PCR recombined *sigK* (qRTBD326-IMV833); lane 2, PCR *skin*^*Cd*^ junction (OBD742-qRTBD326); lane 3, PCR excised form (IMV824-IMV825).
(TIF)

S7 Fig Thin section transmission electron microscopy of 630Δ*erm* and 630Δ*erm* Δ*skin*^*Cd*^ spores. The spores from both strains were purified as described for Fig 8B. The figure shows whole spores from which the panels of Fig 8B were derived. The yellow arrows point to the lamellar inner coat layers, whereas the red arrows point to the more electrondense spore surface layers.
(TIF)

S1 Table List of oligonucleotides. (DOCX)

S2 Table Kinetics of expression of *sigK* and σ^K^ target genes in 630Δ*erm* and 630Δ*erm* Δ*skin*^*Cd*^ strains. Total RNAs were extracted from *C*. *difficile* 630Δ*erm* and 630Δ*erm* Δ*skin*^*Cd*^ strains, after 10 h, 14 h, 18 h, 20 h and 24 h of growth in SM medium. After reverse transcription, specific cDNAs were quantified by qRT-PCR using the DNApolIII gene for normalization.
(DOCX)
