# Supplementary figures and images for: A Recombination Directionality Factor Controls the Cell Type-Specific Activation of σK and the Fidelity of Spore Development in Clostridium difficile
Source: PLoS Genet. 2016 Sep 15;12(9):e1006312. doi: 10.1371/journal.pgen.1006312 (PMC5025042; doi:10.1371/journal.pgen.1006312)

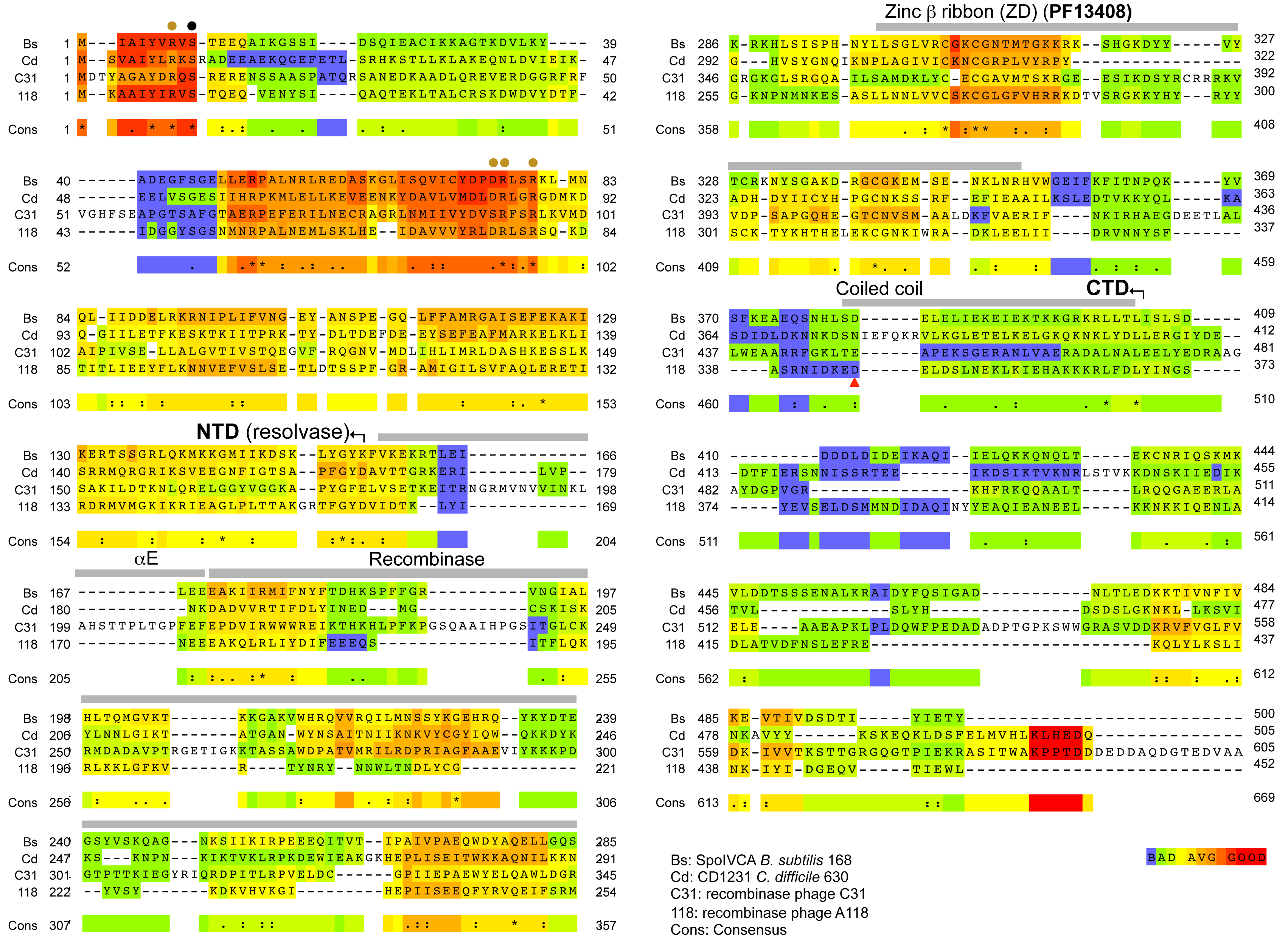

Supplement: S1 Fig — The alignment was generated using T-coffee (www.tcoffee.org). The color code indicates conservation, as indicated by the scale on the lower right corner. The conserved motifs are indicated, as well as the boundaries between the N- (NTD) and C-terminal (CTD) domains of the recombinase. The black dot indicates the catalytic nucleophile (Ser10) and the brown dots additional catalytic residues in CD1231. The position homologous to residue Glu449 in the ϕC31 recombinase is indicated by a red triangle. The E449K substitution in the ϕ C31 protein results in a “hyperactive” recombinase able to recombine attL and attR in the absence of the RDF. (TIF) [file pgen.1006312.s001.tif]

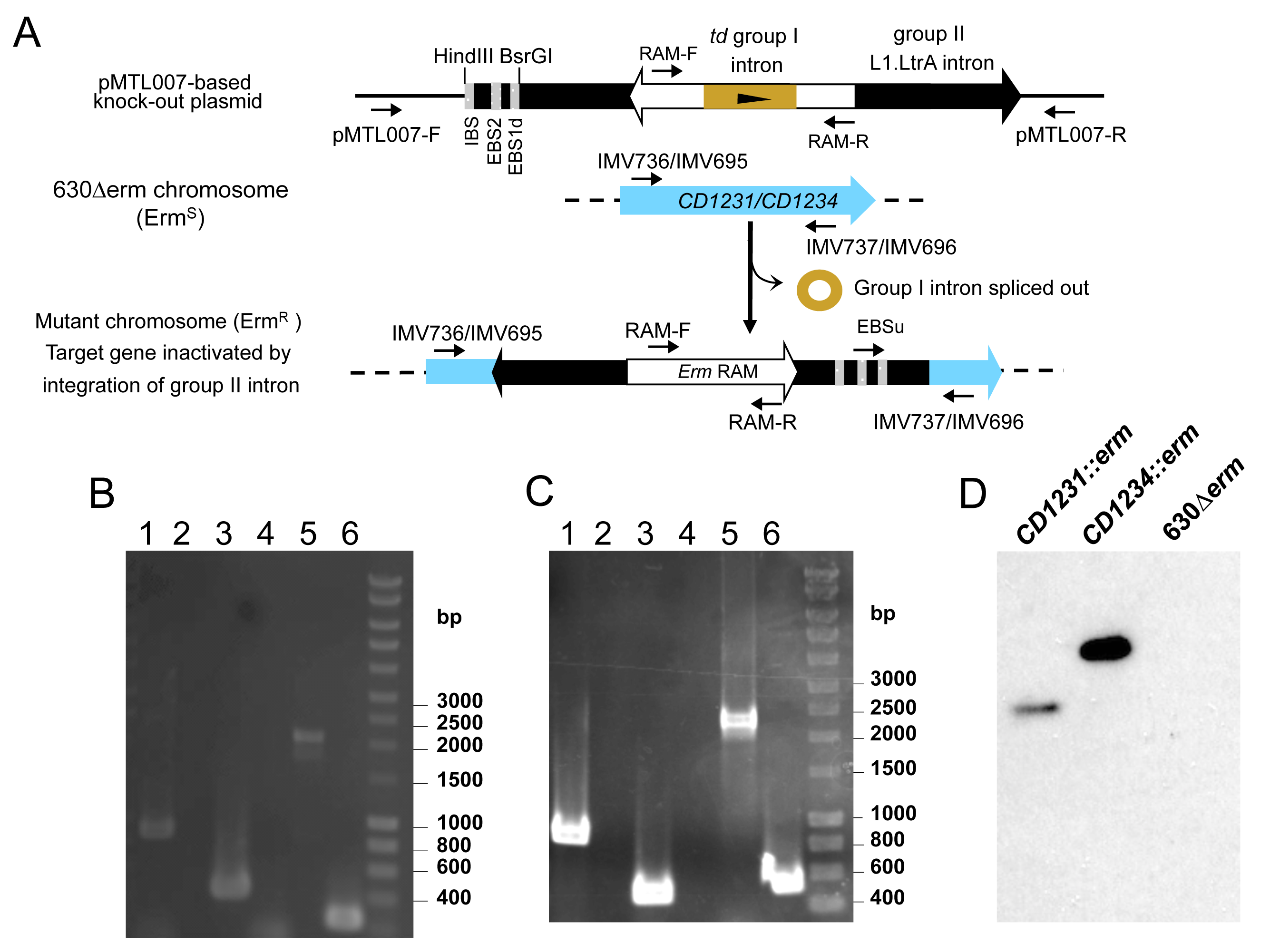

Supplement: S2 Fig — A: schematic representation of gene inactivation by a type II Intron with an associated Retro-transposon-Activated Marker (RAM) [54]. The group II intron (bracket), originally in pMTL007 (top), carries a RAM element interrupting an ermB determinant (white). The intron was retargeted to CD1231 or CD1234 (blue) by altering the IBS, EBS1 and EBS2 sequences (grey and white stripes; top) by overlapping PCR. Splicing out of the td group I intron from the ermB gene in the RAM restores a functional marker allowing positive selection of mutants following intron integration. Primers used to confirm the integration and orientation of the type II intron are also indicated (bottom). B: chromosomal DNA of EmR C. difficile conjugants obtained during CD1231 inactivation by Clostron and of strain 630Δerm were screened by PCR using primer pairs RAM-F/R to confirm splicing out of the group I intron in the mutant (lane 1 and 2). We also performed PCR using chromosomal DNA of strain 630Δerm and of the CD1231 mutant (lane 3 and 4) with the intron primer EBSu and with a primer in CD1231 (IMV737). To verify the integration of the Ll.LtrB intron into the right gene targets, we further performed PCR using chromosomal DNA of strain 630Δerm and of the CD1231 mutant (lane 5 and 6) with two primers flanking the insertion site in CD1231 (IMV737-IMV736). Chromosomal DNA from the 630Δerm strain corresponded to lane 2, 3 and 6 while chromosomal DNA of the CD1231 mutant corresponded to lane 1, 3 and 5. The smart ladder (Eurogentec) was used as a molecular weight marker. C: chromosomal DNA of EmR C. difficile conjugants obtained during CD1234 inactivation by Clostron and of strain 630Δerm were screened by PCR using primer pairs RAM-F/R to confirm splicing out of the group I intron in the mutant (lane 1 and 2). To verify the integration of the Ll.LtrB intron into the right gene targets, we further performed PCR using chromosomal DNA of strain 630Δerm and of the CD1234 mutant with the intron primer EBSu [file pgen.1006312.s002.tif]

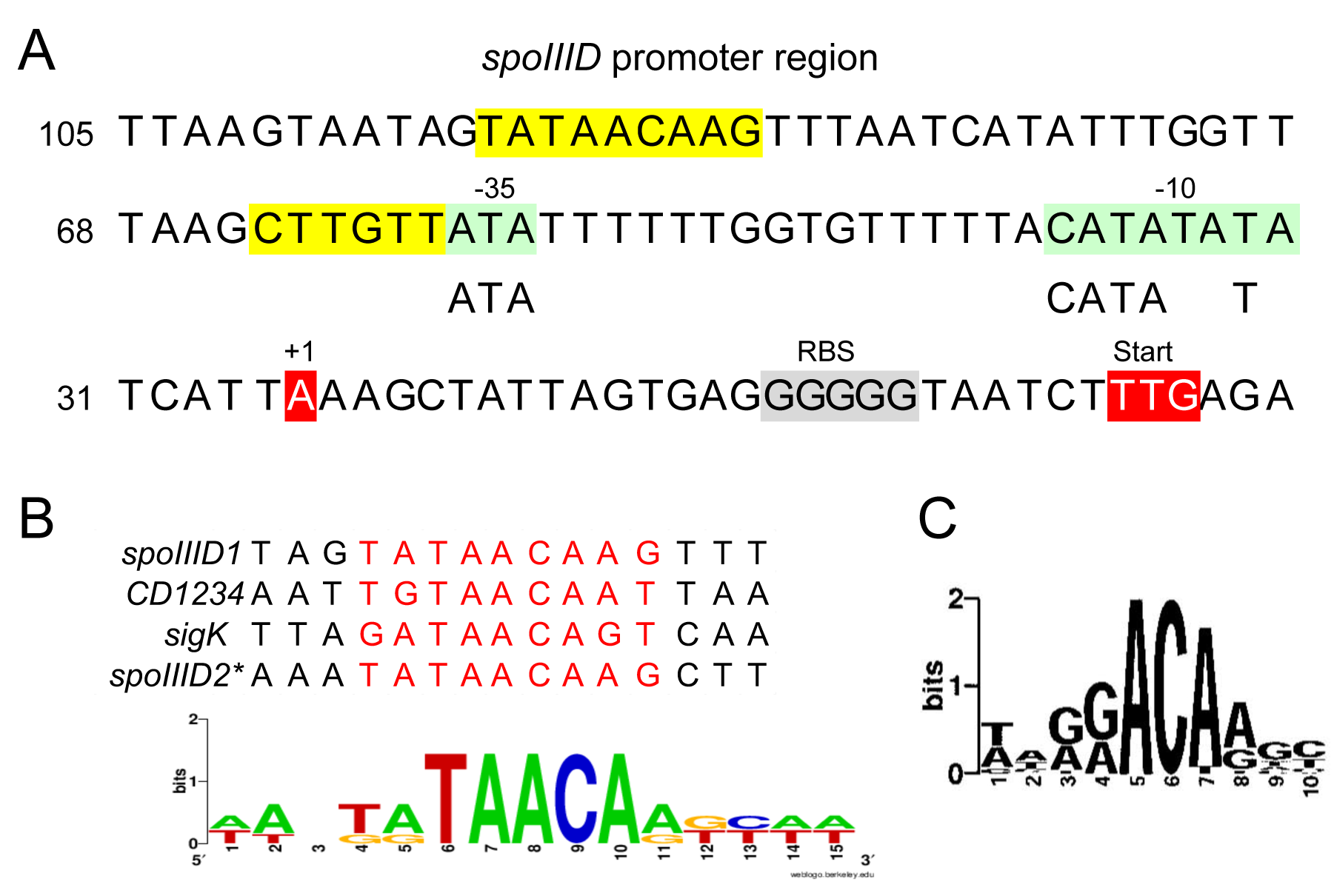

Supplement: S3 Fig — Panel A: The DNA sequence immediately uptream of the coding region of spoIIID is presented. The transcriptional start site (+1, red) as previously mapped [34], the -10 and -35 promoter elements (green) that match the consensus for σE recognition (represented below the sequence), SpoIIID boxes (yellow), and the start codon of spoIIID, are indicated. Panel B corresponds to an alignment of the SpoIIID box identified upstream of CD1234, sigK and spoIIID. The sequence logo was created from on the WebLogo website (http://weblogo.berkeley.edu) using SpoIIID boxes identified in this study. The asterisk means that the sequence of the SpoIIID2 box is in opposite orientation. Panel C shows the SpoIIID box of B. subtilis as described in Eichenberger et al [19]. (TIF) [file pgen.1006312.s003.tif]

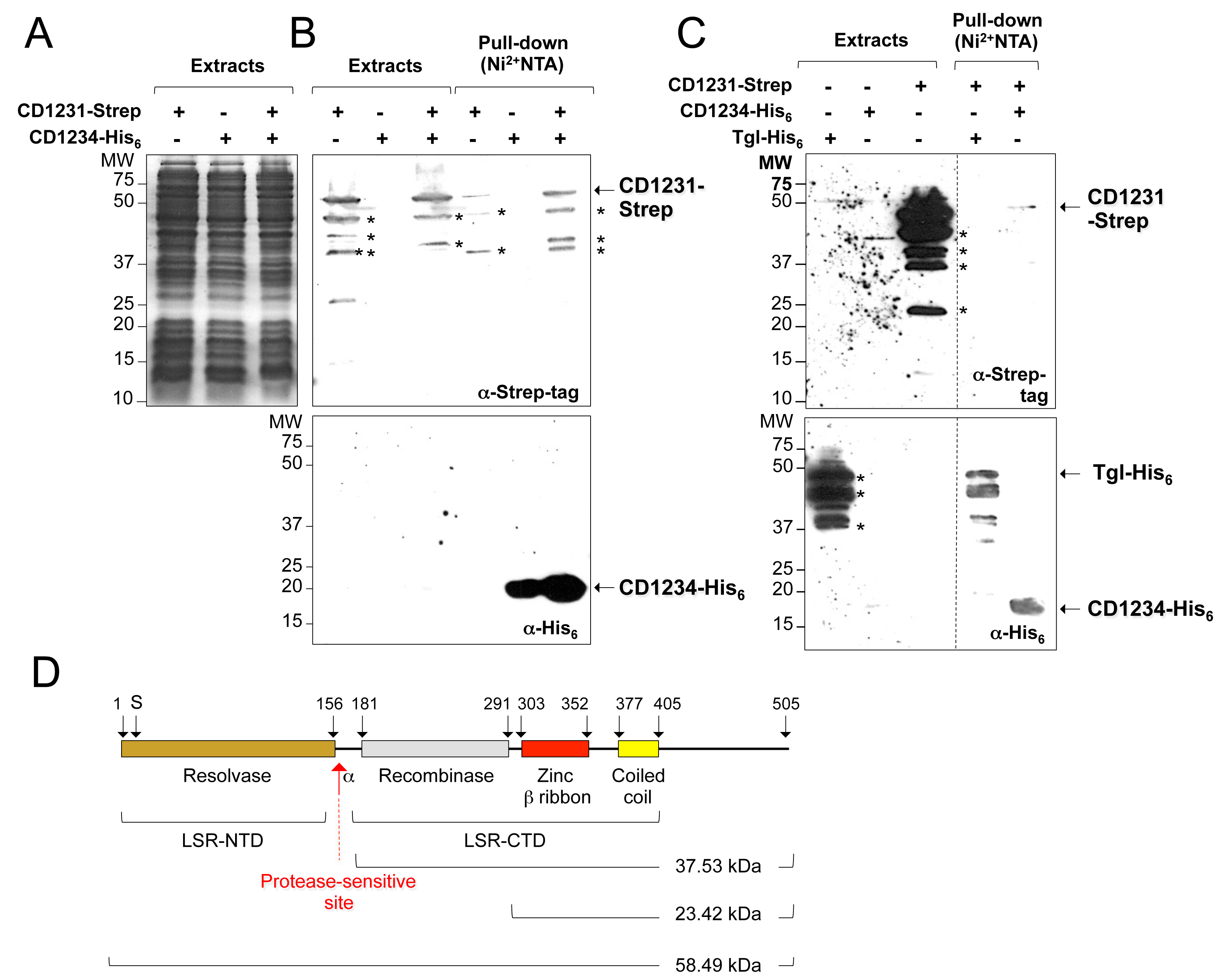

Supplement: S4 Fig — A: whole cell extracts were made from E. coli BL21(DE3) derivatives induced with IPTG to produce CD1231-Strep (expected size 59 kDa), CD1234-His6 (15 kDa), or both (as indicated by the «+ » signs). B: the proteins were detected in the extracts by immunoblotting (first three lanes) using anti-Strep (top) or anti-His6 antibodies (bottom). CD1231-Strep was detected as a species migrating around 60 kDa as indicated (the asterisks represent likely degradation products). CD1234-His6 was detected as a species of about 12 kDa, as indicated. The last three lanes of panel B represent the results of a pull down assay, in which CD1231-Strep (lane 4), CD1234-His6 (lane 5) or the two co-produced proteins (lane 6) were incubated with Ni2+-NTA agarose beads. CD1234-His6 alone (lane 5) binds to the beads. Residual binding of CD1231-Strep to the beads (lane 4) was also detected, but retention of the protein was greatly increased in the presence of CD1234-His6 (lane 6). C: the left side of the panel shows the immnoblot analysis of whole cell extracts prepared from E. coli BL21(DE3) strains producing the indicated proteins (« + » signs) individually. The extracts containing Tgl-His6 [39,40] or CD1234-His6 were incubated with the Ni2+-NTA agarose beads after which the extract containing CD1231-Strep was added. Proteins were eluted, resolved by SDS-PAGE and subject to immunoblot analysis with anti-Strep (top) or anti-His6 antibodies (bottom). The asterisks represent likely degradation products. D: diagram of the domain organization of CD1231 (as in Fig 1B) showing the location of a protease-sensitive site at the end of the NTD, in the recombinases from phages C31 and Bxb1 and from transposon TnpX. The calculated size of the full-lenght protein and of two fragments containing the C-terminal end of the protein (CTD and C-terminal extension) is shown. Fragments of about 37 and 40 kDa most likely containing the C-terminal end of CD1231-Strep may interact with CD1234-His6 (see main text for d [file pgen.1006312.s004.tif]

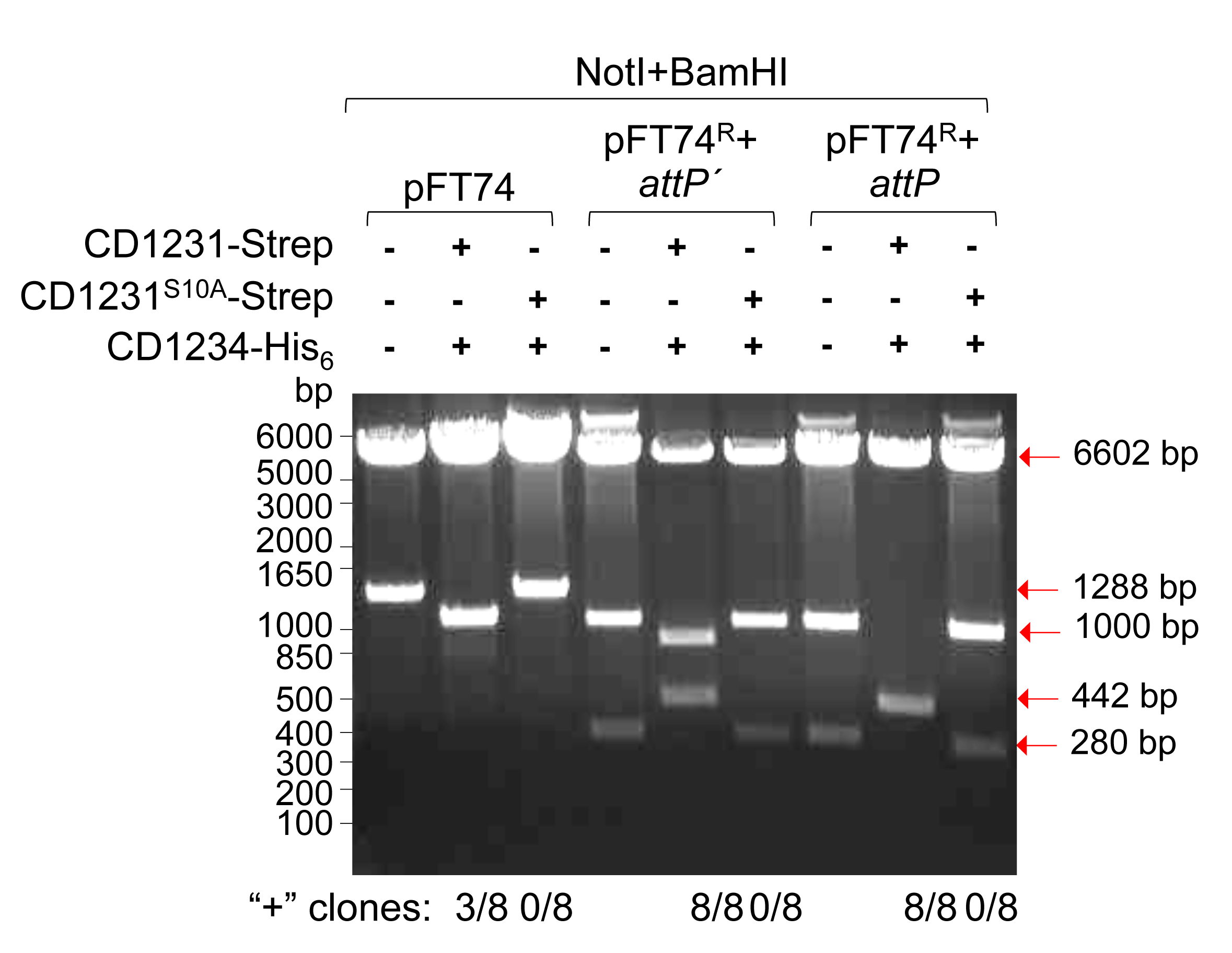

Supplement: S5 Fig — E. coli cells were transformed with plasmids pFT74 and pMS510 and pMS511 corresponding to pFT74R+attP [carrying a recombined sigK gene and containing the attP site in both possible orientations (attP or attP´; see also Fig 7)]. Each E. coli strain containing one of these plasmids was then transformed with plasmids expressing either CD1231-Strep or CD1231S10A-Strep, and CD1234-His6 or both under the control of an IPTG inducible promoter. Plasmids were recovered from E. coli strains and analyzed by digestion with NotI and BamHI. Plasmid DNA from pFT74 and pFT74R+attP were used as controls for non-recombined and recombined sigK, respectively. The size of molecular size marker (in bp) is indicated on the left side of the panels. The “+” sign identifies clones where recombination has occurred (a total of 8 clones were analyzed). (TIF) [file pgen.1006312.s005.tif]

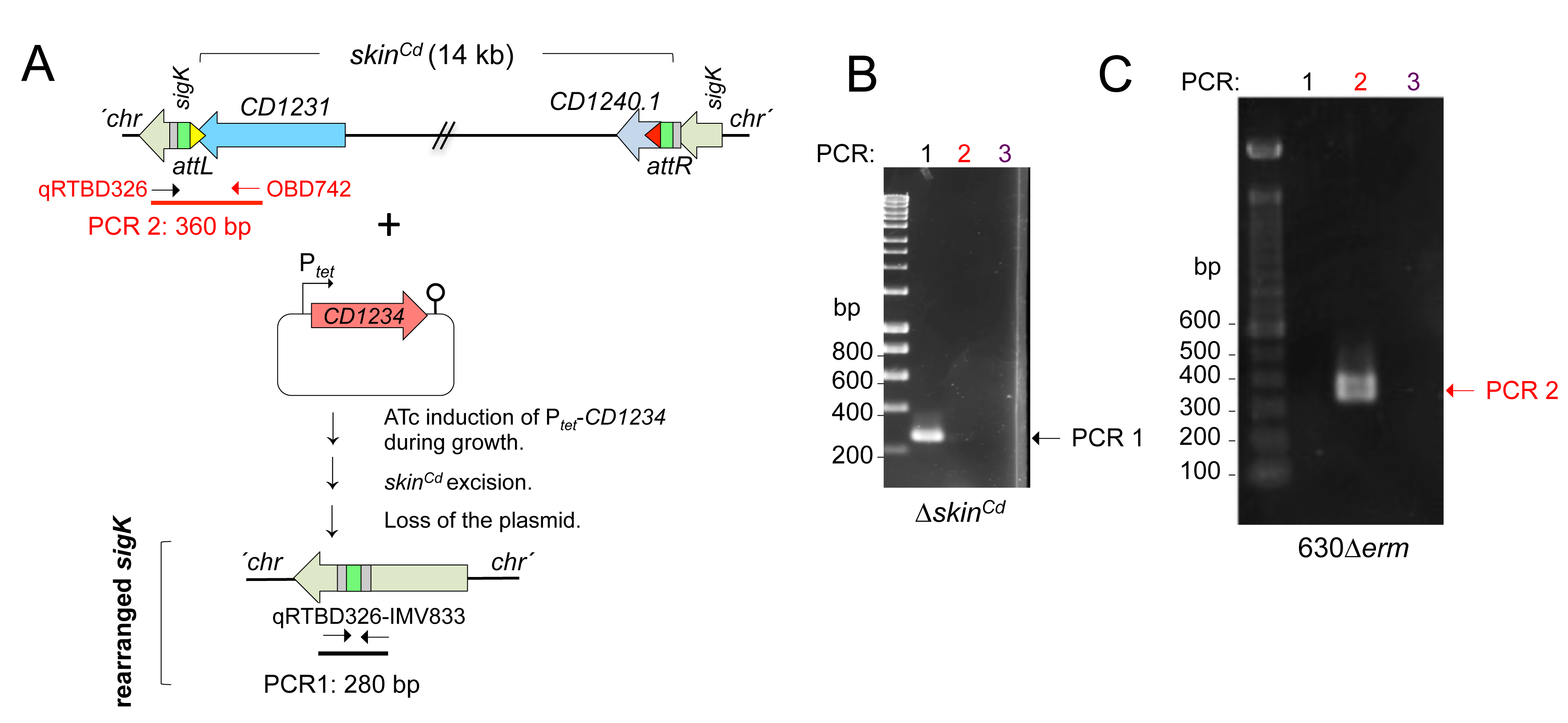

Supplement: S6 Fig — A: strategy of construction of the 630Δerm ΔskinCd strain. A plasmid (pDIA6103-CD1234) carrying the gene CD1234 expressed under the control of a Ptet promoter was transferred into strain 630Δerm by conjugation. After 4 h of growth, CD1234 expression was induced with ATc for 2 h and cells were plated on BHI. The loss of the plasmid was obtained and verified as outlined in the Materials and Methods section. B: verification of the skinCd excision into the chromosome of the strain 630Δerm ΔskinCd after 5 h of growth in TY medium (vegetative cells). Lane 1: PCR recombined sigK (qRTBD326-IMV833); lane 2: PCR skinCd junction (OBD742-qRTBD326); lane 3: PCR excised form (IMV824-IMV825). C: detection of skinCd in strain 630Δerm during vegetative growth in TY medium (4 h). The gel shows the PCR products obtained with the following primer pairs: lane 1, PCR recombined sigK (qRTBD326-IMV833); lane 2, PCR skinCd junction (OBD742-qRTBD326); lane 3, PCR excised form (IMV824-IMV825). (TIF) [file pgen.1006312.s006.tif]

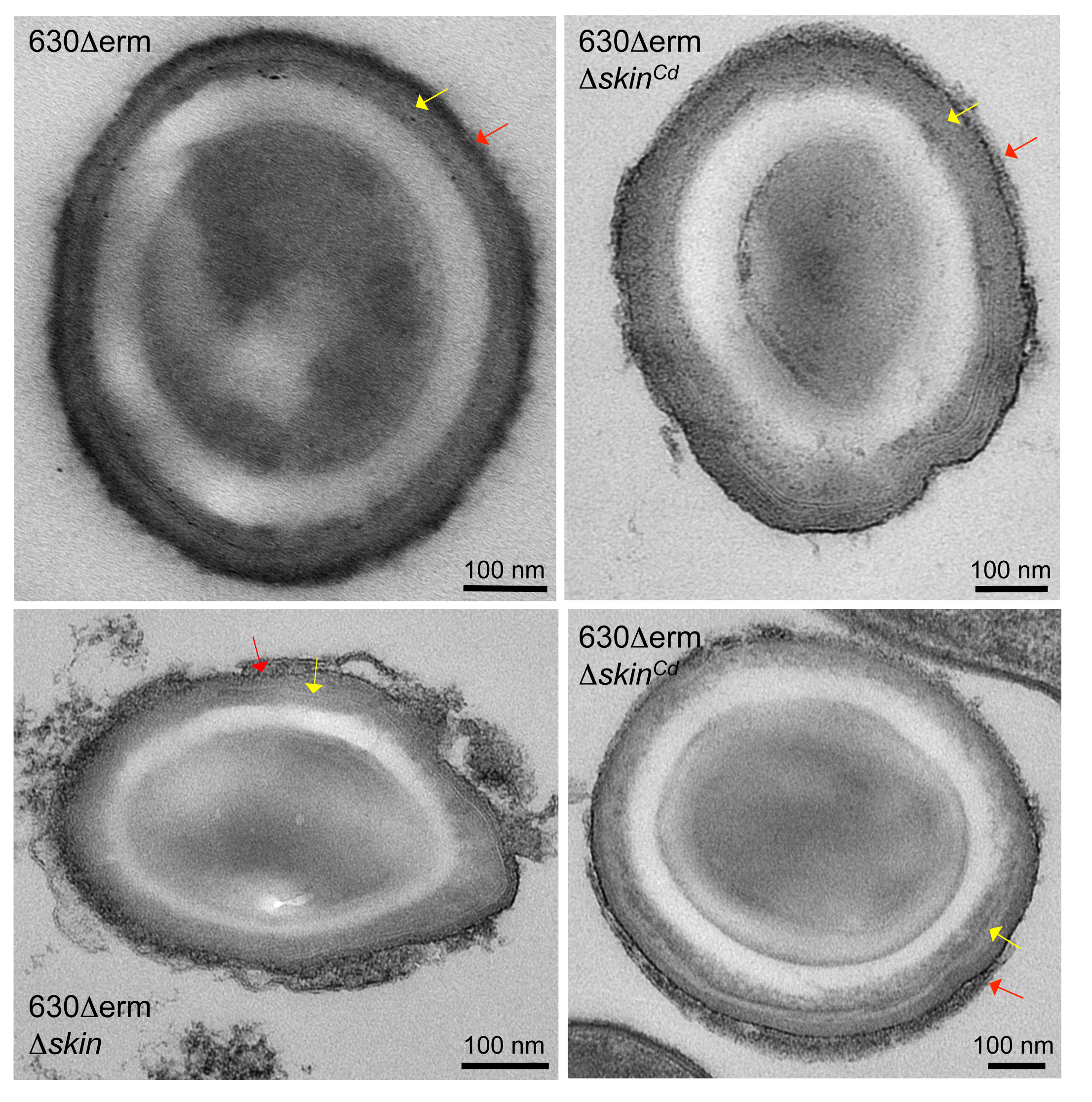

Supplement: S7 Fig — The spores from both strains were purified as described for Fig 8B. The figure shows whole spores from which the panels of Fig 8B were derived. The yellow arrows point to the lamellar inner coat layers, whereas the red arrows point to the more electrondense spore surface layers. (TIF) [file pgen.1006312.s007.tif]
