# Supplementary material for: A Recombination Directionality Factor Controls the Cell Type-Specific Activation of σK and the Fidelity of Spore Development in Clostridium difficile
Source: PLoS Genet. 2016 Sep 15;12(9):e1006312. doi: 10.1371/journal.pgen.1006312 (PMC5025042; doi:10.1371/journal.pgen.1006312)
Supplement: S1 Table — (DOCX) [file pgen.1006312.s008.docx]

**Table S1. Oligonucleotides used in this study**

| **Primer** | Sequence (5´to 3´) | Use/Target |
| --- | --- | --- |
| IMV682 | AAAAAAGCTTATAATTATCCTTATATAACCTTCTGGTG | CD1234-88\|89a-IBS |
|  | CGCCCAGATAGGGTG |  |
| IMV683 | CAGATTGTACAAATGTGGTGATAACAGATAAGTCCTTC | CD1234-88\|89a-EBS1d |
|  | TGTGTAACTTACCTTTCTTTGT |  |
| IMV684 | TGAACGCAAGTTTCTAATTTCGGTTTTATATCGATAGA | CD1234-88\|89a-EBS2 |
|  | GGAAAGTGTCT |  |
| IMV725 | AAAAAAGCTTATAATTATCCTTATATTTCCTTCAAGTG | CD1231-205\|206a-IBS |
|  | CGCCCAGATAGGGTG |  |
| IMV726 | CAGATTGTACAAATGTGGTGATAACAGATAAGTCCTT | CD1231-205\|206a-EBS1d |
|  | CAACTTAACTTACCTTTCTTTGT |  |
| IMV727 | TGAACGCAAGTTTCTAATTTCGGTTAAATATCGATAG | CD1231-205\|206a-EBS2 |
|  | AGGAAAGTGTCT |  |
| EBSu | CGAAATTAGAAACTTGCGTTCAGTAAAC | EBS-universal |
| ErmRAM-F | ACGCGTTATATTGATAAAAATAATAATAGTGGG | PCR *erm* intron |
| ErmRAM-R | ACGCGTGCGACTCATAGAATTATTTCCTCCCG | PCR *erm* intron |
| pMTL007-F | TTAAGGAGGTGTATTTCATATGACCATGATTACG | Sequencing pMTL007 |
| pMTL007-R | AGGGTATCCCCAGTTAGTGTTAAGTCTTGG | Sequencing pMTL007 |
| M13R | CAG GAA ACA GCT ATG AC | Sequencing pMTL84121 |
| M13F | GTTTTCCCAGTCACGAC | Sequencing pMTL84121 |
| SP6 | GATTTAGGTGACACTATAG | Sequencing pGEM-T easy |
| T7 | AATACGACTCACTATAG | Sequencing pGEM-T easy |
| IMV736 | CCCGTGCTGATGAAGAGGC | Insertion intron *CD1231* |
| IMV737 | TTCATGTCTCCACGACCAA | Insertion intron *CD1231* |
| IMV695 | CCGCTCGAGACTAGATACTATATAAGAGGA | 5’XhoI-*CD1234* |
| IMV696 | CGGGATCCTCAACAATTTTATCGCCTT | 3’BamHI-*CD1234* |
| IMV720 | AAGGCCTATAACAAGGGGTGATGTT | 5’ StuI-*CD1234* |
| IMV728 | CCGCTCGAGGCGCTTGAACTTAACCC | 5’XhoI-CD1231 |
| IMV729 | CGGGATCCTACTCTTTATATGTTATTGGTAT | 3’BamHI-CD1231 |
| IMV652 | CCGAATTCAAAATCTATAGACCCAATGA | 5’EcoRI-spoIIID |
| IMV653 | CCGCTCGAGGATCTCAAAGATTACCC | 3’XhoI-spoIIID |
| IMV677 | GGAATTCACTAGATACTATATAAGAGGA | 5’ EcoRI-CD1234 |
| IMV678 | CCGCTCGAGAATTGTTGTAGTATTTTCAT | 3’ XhoI-CD1234 |
| QRTBD325 | CGGAACAGATAAAGAAGGTAATGAA | *sigK* 5‘ *skin* insertion, q-PCR |
| QRTBD326 | TCATCAAGAACATAGTTAGCCTCTG | *sigK* 3‘ *skin* insertion, q-PCR |
| DNA-*polIII* | TCCATCTATTGCAGGGTGGT | qRT-PCR or q-PCR |
| DNA-*polIII* | CCCAACTCTTCGCTAAGCAC | qRT-PCR or q-PCR |
| PB124 | TGCTGCTAATGCAGTGAAAAA | q-PCR CD1233 |
| PB125 | CCCTCTGCACCCTGTTTAAT | q-PCR CD1233 |
| OBD742 | CATTTATTGAAAGGTCCAACA | CD1231, PCR *skin* junction |
| IMV833 | TTCAACGGAAGATCAGGATG | PCR *sigK* |
| IMV824 | CCATGATTCAGATTCCCTTG | PCR *skin* circularized |
| IMV825 | AAAAGTGTTTTGAATGGGGATT | PCR *skin* circularized |
| LS100 | CACCACCTCAATGTGGAAAA | qRT-PCR 5' *spoIIIAA* |
| LS101 | GCTCCTGCTATCTCATTACGC | qRT-PCR 3' *spoIIIAA* |
| LS102 | GGGCCATAGTGGTAGCAAAA | qRT-PCR 5' *CD0126-spoIIID* |
| LS103 | TGGCAAGGGATGGATTTATT | qRT-PCR 3' *CD0126* |
| LS106 | CCCCAAAGTGGTTCAGGTAA | qRT-PCR 5' *spoIVA* |
| LS107 | TGCCCTAAAGCTCCTTCAAC | qRT-PCR 3' *spoIVA* |
| LS143 | CAGGCCCAAATGGTAGAAAA | qRT-PCR 5' *CD1433-cotE* |
| LS144 | GAAGGCATTCCAGCATTCTC | qRT-PCR 3' *CD1433* |
| LS155 | GAAAAACCCTTAACCCCTGA | qRT-PCR 5' *CD1230-sigK* |
| LS156 | TCATCCTGATCTTCCGTTGA | qRT-PCR 3' *CD1230-sigK* |
| \| LS352 \| \| --- \| | AGGCAAAAGGAAAGCATGAA | qRT-PCR 5’ *CD1231* |
| LS353 | TGCAAATCACTATTCCTGCAA | qRT-PCR 3’*CD1231* |
| LS354 | AACAAGGGGTGATGTTATGAAAA | qRT-PCR 5’ *CD1234* |
| LS355 | TTTTCTTCATCAGATATACGTGGA | qRT-PCR 3’*CD1234* |
| LS143 | GAAGGCATTCCAGCATTCTC | qRT-PCR *CD1433-cotE* |
| LS144 | CAGGCCCAAATGGTAGAAAA | qRT-PCR *CD1433-cotE* |
| LS147 | CCAATGTCCTGACCAAATGA | qRT-PCR *CD0551-sleC* |
| LS148 | CTGCCCAGAAGAACCAACTT | qRT-PCR *CD0551-sleC* |
| LS158 | TTAAGGCTGCTGGACTTGGT | qRT-PCR *CD0598-cotCB* |
| LS159 | AGTTACCGAATCGCCAAAGA | qRT-PCR *CD0598-cotCB* |
| LS164 | CGTTGACGAAATAGCACCTG | qRT-PCR *CD3580* |
| LS165 | TGGGGAAATACAATAGAAGCGTA | qRT-PCR *CD3580* |
| LS166 | TTGATATGGCCCACAAAGAA | qRT-PCR *CD1133* |
| LS167 | CACTTTCTTGATATGCACACTTTTT | qRT-PCR *CD1133* |
| LS206 | GCGGAGGAGTACTTTCTGGA | qRT-PCR *CD0332-bclA1* |
| LS207 | GCTGATTGCCCATTTCGTAT | qRT-PCR *CD0332-bclA1* |
| PCDsigK5’ Fw | GCTGCGGCCGCTGACTGATACTTTTG |  |
| CDsigK5’ Rev | CACTTTTCTTAAATCACTAGCTATAGAAATTGATGA |  |
| CDsigK3’ Fw | TCATCAATTTCTATAGCTAGTGATTTAAGAAAAGTG | P-*_CD1231_*-SNAP fusion |
| CDsigK3’EcoRI Rev | GCTGAATTCTTAACTTTCTTGAACAAG | P-*_CD1231_*-SNAP fusion |
| recFP-Fw | GTGTTATCAGAATTCCATGATTCAGATTCC |  |
| SigK-SNAP Rev | agctgcggatccaccaccaccaagACTTTCTTGACAAGCTCTTTTTC |  |
| CD1231 Fw | GTTACCATGGCTGTTGCCATATAC |  |
| CD1231 Rev | ATGTTATTGGTCGACCTGGTCTTCATG |  |
| CD1234 Fw | GTATAAGACCCATGGATGAGTTCATAT |  |
| CD1234 Rev | TTCTCTATTCTCGAGACTTTCTACTGT |  |
| CD1231 S10A Fw | ATGAGTGTTGCCATATACTTAAGAAAAGCCCGTGC |  |
| CD1231 S10A Rev | GCACGGGCTTTTCTTAAGTATATGGCAACACTCAT |  |
| SNAP-R | CCCAAGCTTTTACCCAAGTCCTGGTTTCCCCAAACG |  |
| OBD522 | ATCTGTAGGAGAACCTATGGGAAC | Intron probe |
| OBD523 | CACGTAATAAATATCTGGACGTAAAA | Intron probe |
