# Supplementary material for: A Recombination Directionality Factor Controls the Cell Type-Specific Activation of σK and the Fidelity of Spore Development in Clostridium difficile
Source: PLoS Genet. 2016 Sep 15;12(9):e1006312. doi: 10.1371/journal.pgen.1006312 (PMC5025042; doi:10.1371/journal.pgen.1006312)
Supplement: S2 Table — Total RNAs were extracted from C. difficile 630Δerm and 630Δerm ΔskinCd strains, after 10 h, 14 h, 18 h, 20 h and 24 h of growth in SM medium. After reverse transcription, specific cDNAs were quantified by qRT-PCR using the DNApolIII gene for normalization. (DOCX) [file pgen.1006312.s009.docx]

**Table S2.** **Expression of *sigK* and σ^K^ targets in 630∆*erm* and 630∆*erm* ∆*skin^Cd^* strains**

| gene | Ratio of expression (∆*skin^Cd^/*630∆*erm*) | | | | |
| --- | --- | --- | --- | --- | --- |
|  | 10 h | 14 h | 18 h | 20 h | 24 h |
| *sigK* | 4 | 15 | 7 | 3 | 2 |
| *cotE* | 2.8 | 46 | 16 | 8.5 | 1.5 |
| *cotBC* | 4 | 360 | 180 | 90 | 1 |
| *sleC* | 2.6 | 27 | 11 | 4 | 2 |
| *cdeC* | 2.6 | 32 | 20 | 15 | 2 |
| *bclA1* | 3.3 | 10 | 8.5 | 3.8 | 2 |
| *bclA3* | 5.5 | 23 | 38 | 17 | 2 |
